# A new perspective on special effective interventions for metabolic syndrome risk factors: a systematic review and meta-analysis

**DOI:** 10.3389/fpubh.2023.1133614

**Published:** 2023-07-14

**Authors:** Haonan Wang, Yinghong Dai, Sike Huang, Siyu Rong, Yufei Qi, Bin Li

**Affiliations:** ^1^National Clinical Research Center for Geriatric Disorders, Department of Geriatrics, Xiangya Hospital, Central South University, Changsha, China; ^2^Department of Exercise Physiology, Beijing Sport University, Beijing, China; ^3^Department of Physical Education and Research, Central South University, Changsha, China; ^4^Xiangya School of Medicine, Central South University, Changsha, China; ^5^Sports and Art Institute, Hunan University of Chinese Medicine, Changsha, China

**Keywords:** metabolic syndrome, moderate-to-vigorous physical activity, intermittent fasting, combined intervention, meta-analysis

## Abstract

Metabolic syndrome (MetS) has the largest global burden of all noncommunicable diseases. Owing to the clinical heterogeneity of MetS, wide variations have been reported in the efficacy of moderate-to-vigorous physical activity (MVPA) and intermittent fasting (IF) for improving MetS. We searched five databases for randomized controlled trials published through December 2021, and 372 participants from 11 studies were included in this meta-analysis. Compared with MVPA alone, IF combined with MVPA had a more significant effect on improving body mass and levels of fasting blood glucose and high-density lipoprotein cholesterol; however, it was ineffective in improving triglycerides level, systolic blood pressure, and diastolic blood pressure. Subgroup analysis showed that, except for blood pressure, time-restricted fasting combined with MVPA had a better effect than alternate-day fasting with MVPA. Meanwhile, when the intervention lasted longer than 8 weeks, the effect of the combined intervention was significantly better than that of MVPA alone. This finding provides a basis for clinicians to manage the health of overweight individuals. This study also showed that Caucasians may be more suitable for the combined intervention than Asians. And the combined intervention may provide a preventive effect for MetS risk factors in healthy populations, although this may be due to the small sample size. In general, this study provides a novel perspective on special interventions for MetS traits.

## 1. Introduction

Metabolic syndrome (MetS) poses a great risk to human health as a chronic non-communicable disease (1). MetS is a multiple risk factor consisting of several metabolism-related risk factors, including abdominal obesity, insulin resistance, dyslipidemia, and hypertension (2–4), and is known to have increased incidence of type 2 diabetes (T2D) and cardiovascular disease (CVD) (4–7).

Numerous studies have confirmed that behavioral changes in healthy lifestyles, such as physical activity (PA) engagement and calorie restriction (CR), are major contributors to reducing MetS risk factors (8, 9). PA engagement and CR are generally recommended by clinicians as the preferred interventions (6, 9). According to the intensity level, PA can be divided into low, medium, and high intensity exercises. Notably, although a number of studies have shown that low-to-moderate intensity exercise provides significant health benefits (10, 11), moderate-to-vigorous intensity physical activity (MVPA) is recommended by The Physical Activity Guidelines for Americans owing to its better effect (12). MVPA has a metabolic equivalent of task (MET) value of 3 or greater, where MET refers to the rate of energy expenditure in a comfortable state (13). Types of MVPA include, but are not limited to, walking briskly, dancing, jogging, running, etc. (12, 13). MVPA is suggested in adults with metabolic abnormalities, and the benefits usually outweigh the risks among patients without contraindications, according to the World Health Organization 2020 guidelines (14). Meanwhile, as one of the most common ways to restrict calories, intermittent fasting (IF) has been shown to improve metabolism in patients with MetS (6, 15–18). Comparing to traditional CR, IF is more effective at improving metabolic indicators, including reducing body weight, waist circumference, and adiposity (19, 20). IF can be divided into many types, among which time-restricted fasting (TRF) and alternate-day fasting (ADF) have received much attention recently owing to their excellent effectiveness (21–24). TRF is a feeding pattern that restricts eating to specific hours every day without calorie restriction and allows fasting times of >12 h, whereas ADF is a dietary pattern defined as 36 h of strict fasting (“fast days”) followed by 12 h of *ad libitum* feeding (“feast days”) (25).

Although MVPA and/or IF have been reported to be beneficial for individuals with MetS risk factors in several studies, it remains unclear whether a combined intervention would have a better effect than a single intervention. In this study, by reviewing and meta-analyzing reliable results from previous studies, we evaluated and compared the benefits of MVPA combined with IF with a single intervention on the MetS risk factors. This study provides a novel perspective on specific effective intervention for the MetS risk factors.

## 2. Materials and methods

This study was conducted in accordance with the PRISMA 2020 guidelines (26). The PRISMA checklist can be found in Supplementary Table 1. This study was registered in PROSPERO on January 19, 2022, under the registration number CRD42022297776 (27).

### 2.1. Data sources and search strategies

Five English databases (PubMed, Web of Science, Cochrane Library, Embase, and Scopus) were searched from their inception dates (January 1, 1945, January 1, 1950, January 1, 1996, January 1, 1975, and January 1, 2004) to December 31, 2021. The search terms included: “Exercise,” “Physical Activity,” “Aerobic Exercise,” “Endurance Training,” “Resistance Training,” “High-Intensity Interval Training,” “Intermittent Fasting,” “Time Restricted Feeding,” “Alternate-day Fasting,” “Metabolic Syndrome,” and “Metabolic Disease.” We also manually searched for published reviews and their references to identify other studies that met the criteria. Examples of search strategies for the three databases can be found in Supplementary Table 2.

### 2.2. Study selection and eligibility criteria

Three authors (HW, YD, and BL) selected the eligible studies independently by reading the titles, abstracts, and full texts of the publications. The inclusion criteria were as follows: (1) randomized controlled trials (RCT); (2) studies published before December 2021; (3) the participants included healthy and obese adults, both male and female; (4) the methods included a description of the IF protocol and a PA program during the trial period; and (5) studies reporting the effect of IF combined with MVPA on the primary risk factors for MetS and the outcomes included body mass (BM), fasting blood glucose (FBG) levels, triglycerides (TG) levels, high-density lipoprotein cholesterol (HDL-C) levels, systolic blood pressure (SBP), and diastolic blood pressure (DBP).

The exclusion criteria were as follows: (1) non-RCT studies; (2) non-human studies; (3) reviews, case studies, surveys, abstracts, conference papers, or repeated studies; (4) studies in elite or professional athletes; and (5) interventions that did not include IF alone or MVPA alone.

### 2.3. Data extraction

Three authors (HW, SH, and SR) extracted the data by screening abstracts and full texts separately. The extracted information included the author, publication year, number of participants, baseline characteristics in the intervention and control groups, mean age, IF and MVPA intervention measures, outcomes, and study duration.

### 2.4. Quality assessment of studies

Three authors (HW, YD, and SH) evaluated the quality of the included studies using the Cochrane Collaboration Risk of Bias (ROB2.0) to assess the risk of bias, as recommended (28). The Cochrane tool analyzed the following: randomization process, deviations from the intended interventions, missing outcome data, measurement of the outcome, selection of the reported result, and overall bias. Risk of bias were classified as “high,” “low,” or “some concerns” when it was unclear whether a specific bias was present. Two authors (BL and YQ) resolved the disagreements in the evaluation process.

### 2.5. Meta-analysis of data

STATA ver. 16.0 was used to analyze the heterogeneity of the studies and calculate the $I^{2}$ statistic. To calculate the effect size of each study, we used the mean change and S.D. of the outcome measures from baseline to the end of the intervention in the control and intervention groups (29). Outcome indicators were merged with the effect value Z, weighted mean difference ( W.M.D ), and 95% confidence interval ( 95%CI ). We used forest plots to visualize the results of the meta-analysis, and funnel plots and Egger’s test to assess publication bias. We also performed a sensitivity analysis of the amalgamation results of the $I^{2}$ statistic to explore the sources of heterogeneity and determine the corresponding reasons.

## 3. Results

### 3.1. Selection and identification of studies

**Figure 1:**
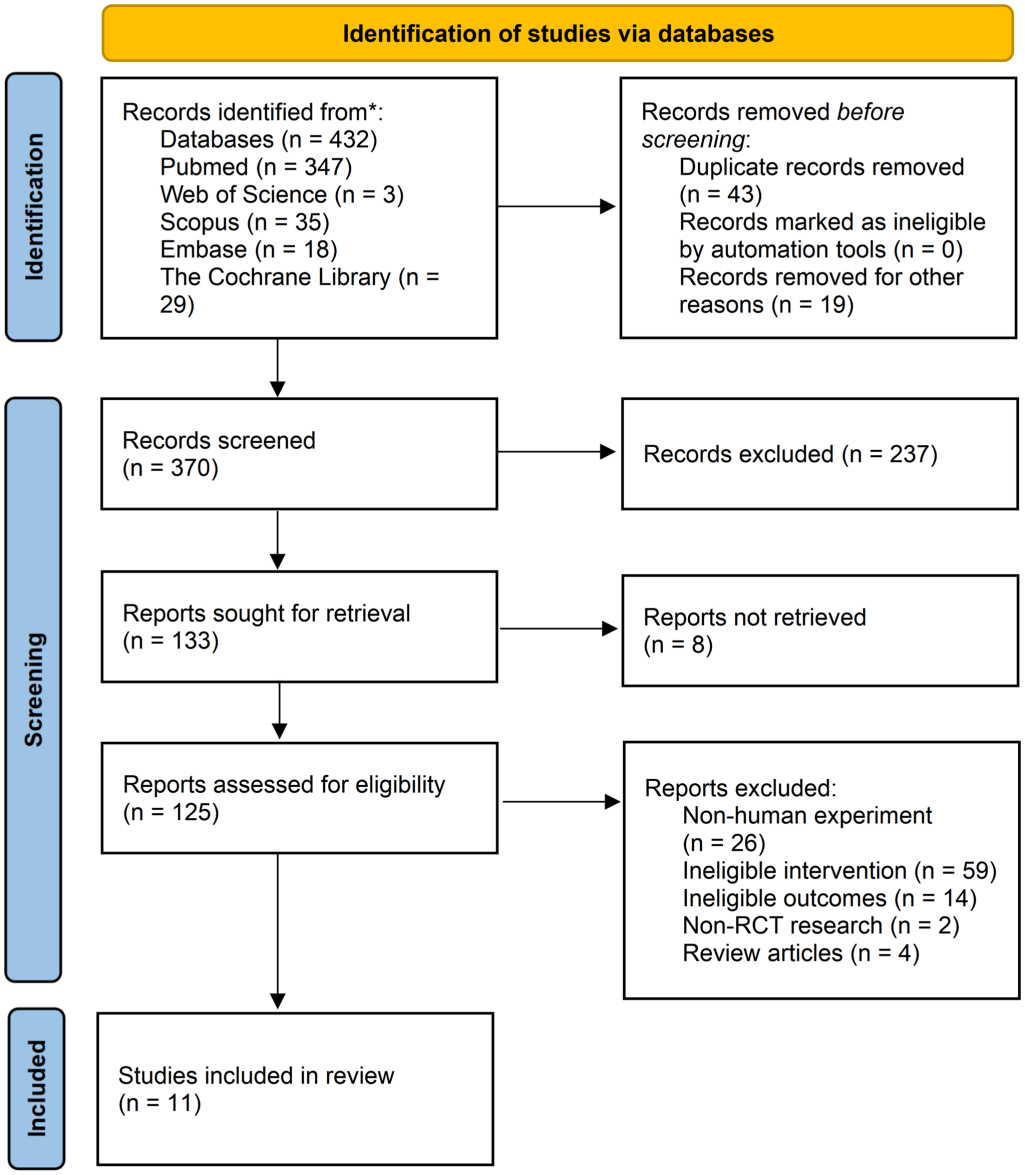
Flow diagram of studies selection. ^*^ Reporting the number of records identified from each database.

In total, 432 records were selected according to the search strategy used in our study (Supplementary Table 2). After eliminating duplicate literature and similar studies, the titles and abstracts of 370 studies were manually reviewed. In the next screening stage, 237 records were excluded based on the Population, Intervention, Comparison, Outcomes and Study (PICOS) criteria. Starting with 133 studies, we checked the availability of full text and further removed eight studies. A total of 125 records were assessed through a strict qualification review of the full text. Finally, 372 participants in 11 studies were included (30–40), which including three multi-arm parallel-group randomized trials that were suitable for comparison between the combination group and the MVPA or IF group (30–32) (Figure 1).

### 3.2. Characteristics of studies

All the included studies compared a combination of IF and MVPA intervention with a control group of IF or MVPA intervention alone. With a total sample size of 372 participants, 154 were in the combination group, 154 in the MVPA group, and 64 in the IF group. The average age of the participants ranged from 22.4 to 43.3 years. The types of MVPA included endurance training (30), aerobic exercise (31, 32), resistance training (31–35, 37, 38), high-intensity interval training (36), and physical activity of the undefined type (39, 40), whereas the types of IF were ADF (30–32) and TRF (33–40). The intervention duration varied from 4 to 48 weeks. The general characteristics of the included studies are summarized in Supplementary Table 3.

### 3.3. Bias risk assessment

The results of the Cochrane’s risk of bias assessment showed that the overall bias in most of the literature was low, whereas three studies raised some concerns and the bias in one study was high (Supplementary Figure 1A). All studies showed a low risk of bias in “measurement of the outcome,” and only one study had some concerns due to insufficient information in the “randomization process” and “selection of the reported result.” However, two studies had incomplete results with missing data. Owing to their experimental design, two papers presented a high risk of deviation from the intended interventions (Supplementary Figure 1B).

### 3.4. Effect on body mass

**Figure 2:**
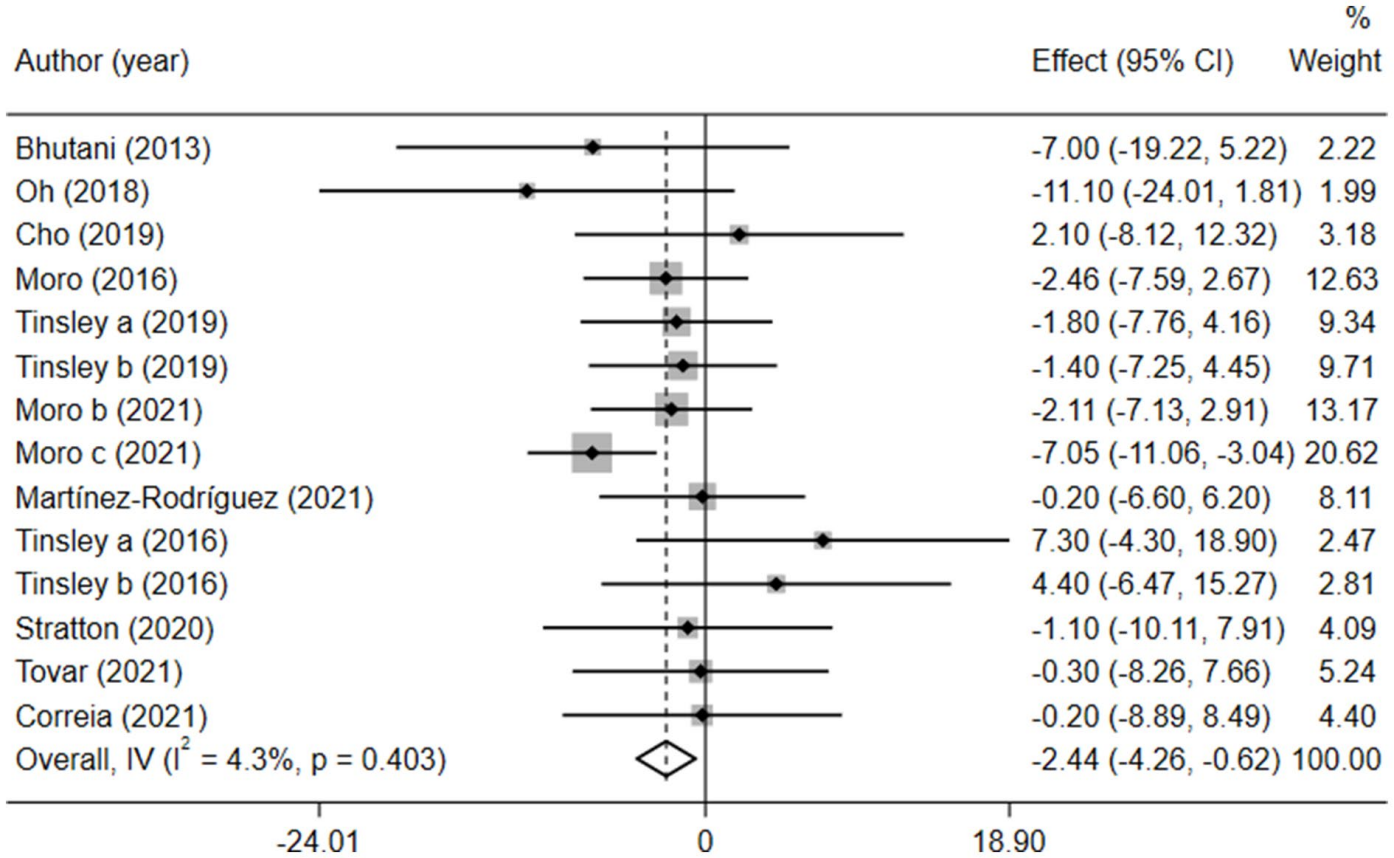
Forest plot of the overall meta-analysis for the effects on BM levels. Each study-specific estimate is represented by a small solid diamond with adjoining horizontal lines which represent the 95% confidence intervals. The size of the gray square surrounding the study-specific estimates represents the weight of each study in the meta-analysis. The diamond with an ascending dashed line from its upper point is the summary estimate. The width of diamond represents the 95% confidence intervals of the summary estimate.

Obesity is a high-risk factor for insulin resistance and T2D (7) and is also a typical clinical trait of MetS. Therefore, BM is often regarded as a direct indicator of MetS. We analyzed 308 participants from 11 studies based on BM and found that BM was significantly lower in the combination group than in the MVPA group [weighted mean difference (WMD) = −2.44, 95% CI −4.26 to −0.62, *p* = 0.009]. This finding strongly suggested that the combination therapy was more suitable for obese patients with MetS (Figure 2). Sensitivity analysis (Supplementary Figure 2) revealed that the 2021 study by Moro et al. (35) was a source of heterogeneity. Meanwhile, publication bias between the included studies was assessed using a funnel plot (Supplementary Figure 3) and Egger’s test (Supplementary Figure 4), which showed no significant publication bias (*p* = 0.110).

**Figure 3:**
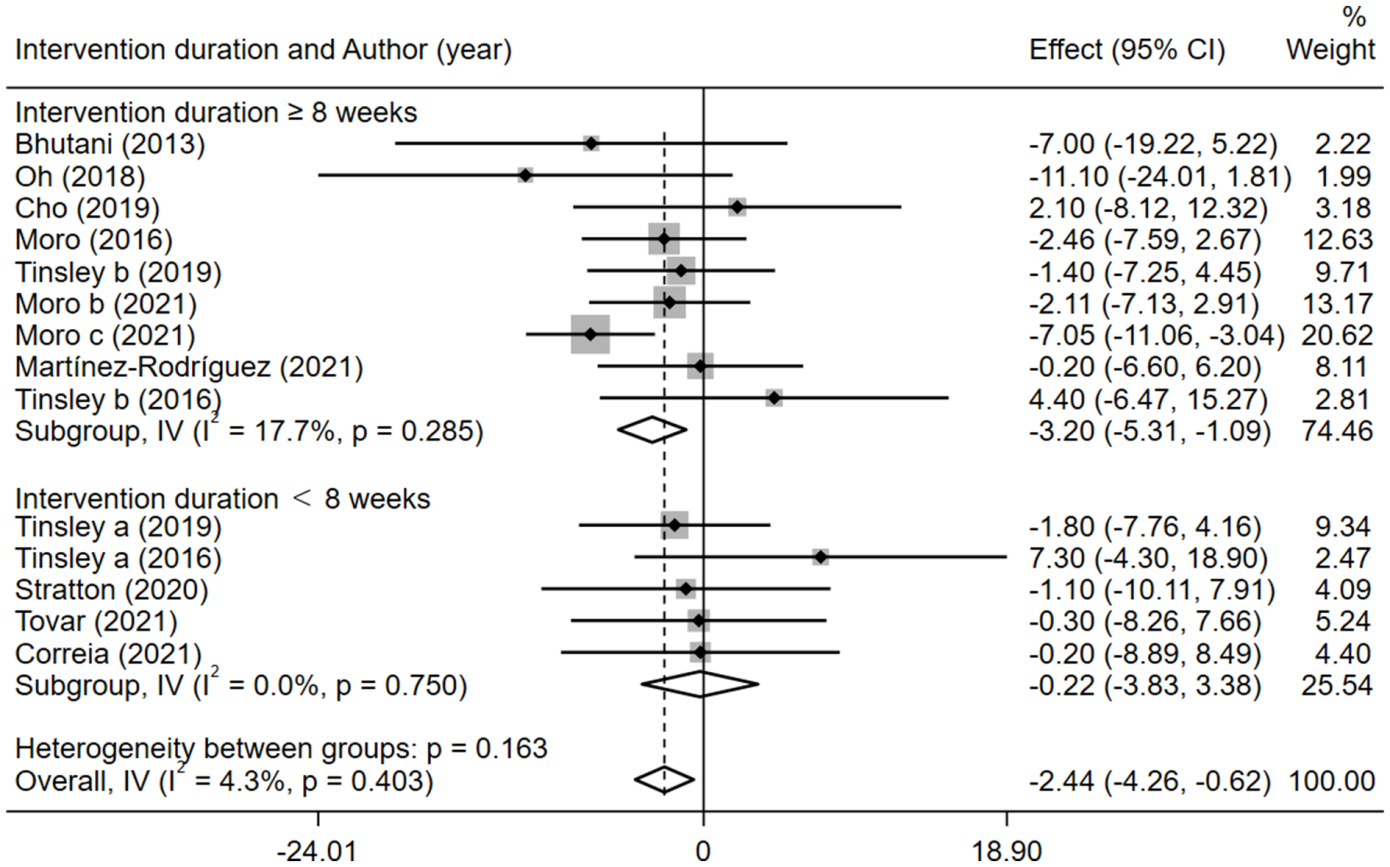
Forest plot of subgroup analysis for the effects on BM with different intervention duration. Each study-specific estimate is represented by a small solid diamond with adjoining horizontal lines which represent the 95% confidence intervals. The size of the gray square surrounding the study-specific estimates represents the weight of each study in the meta-analysis. The diamond with an ascending dashed line from its upper point is the summary estimate. The width of diamond represents the 95% confidence intervals of the summary estimate.

**Figure 4:**
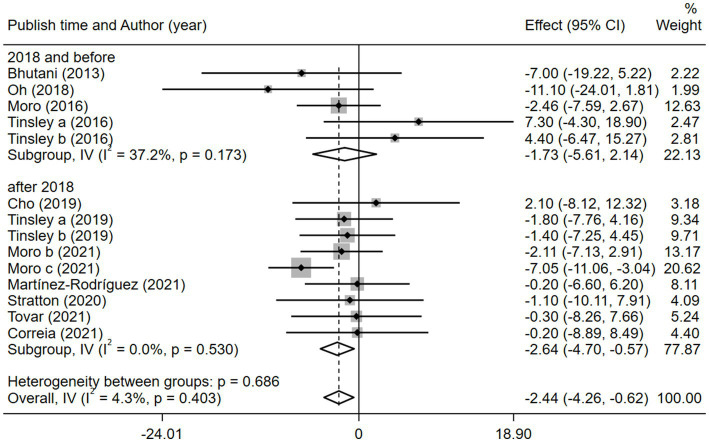
Forest plot of subgroup analysis for the effects on BM with different publication time. Each study-specific estimate is represented by a small solid diamond with adjoining horizontal lines which represent the 95% confidence intervals. The size of the gray square surrounding the study-specific estimates represents the weight of each study in the meta-analysis. The diamond with an ascending dashed line from its upper point is the summary estimate. The width of diamond represents the 95% confidence intervals of the summary estimate.

**Figure 5:**
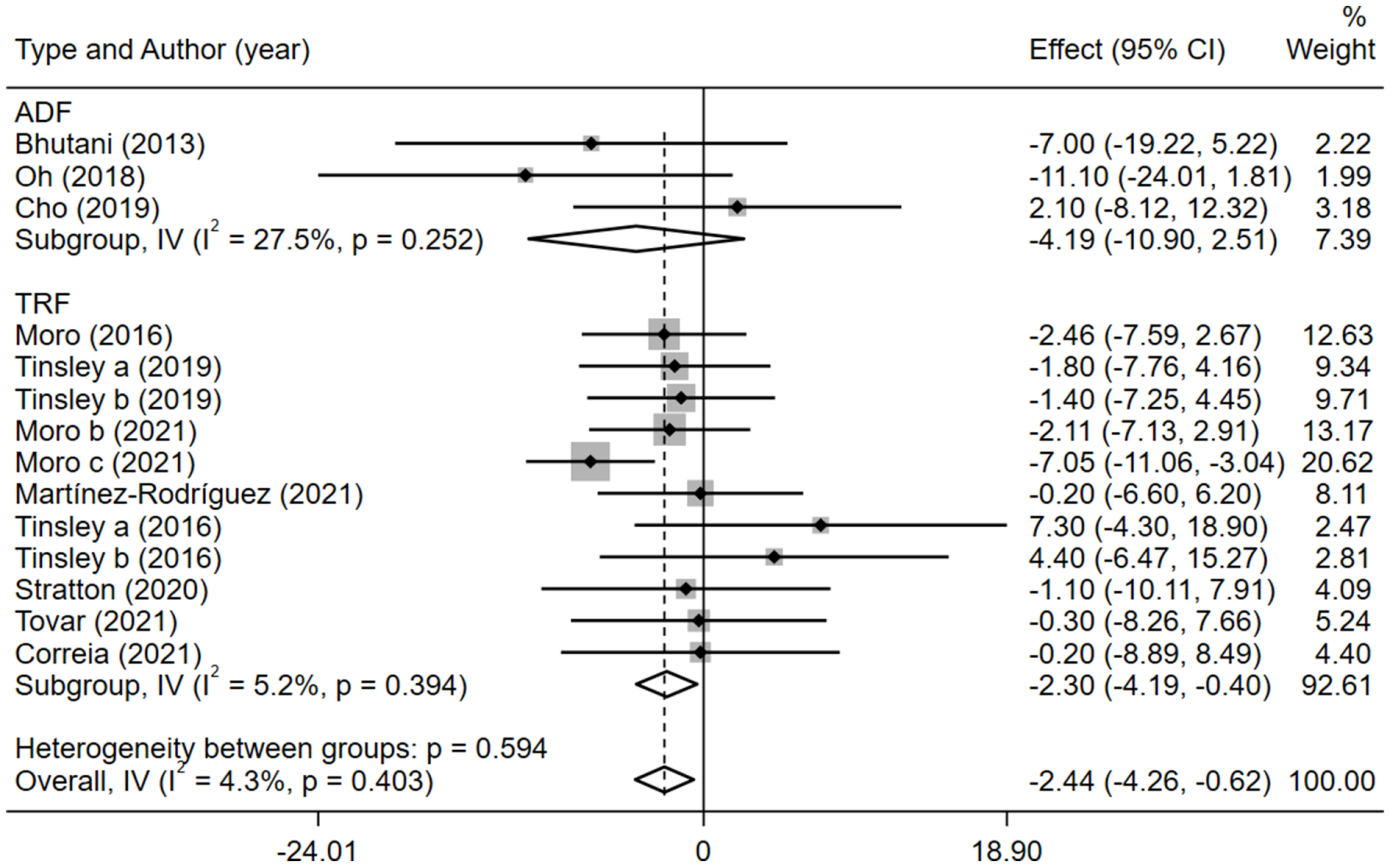
Forest plot of subgroup analysis for the effects on BM with different IF subtypes. Each study-specific estimate is represented by a small solid diamond with adjoining horizontal lines which represent the 95% confidence intervals. The size of the gray square surrounding the study-specific estimates represents the weight of each study in the meta-analysis. The diamond with an ascending dashed line from its upper point is the summary estimate. The width of diamond represents the 95% confidence intervals of the summary estimate.

**Figure 6:**
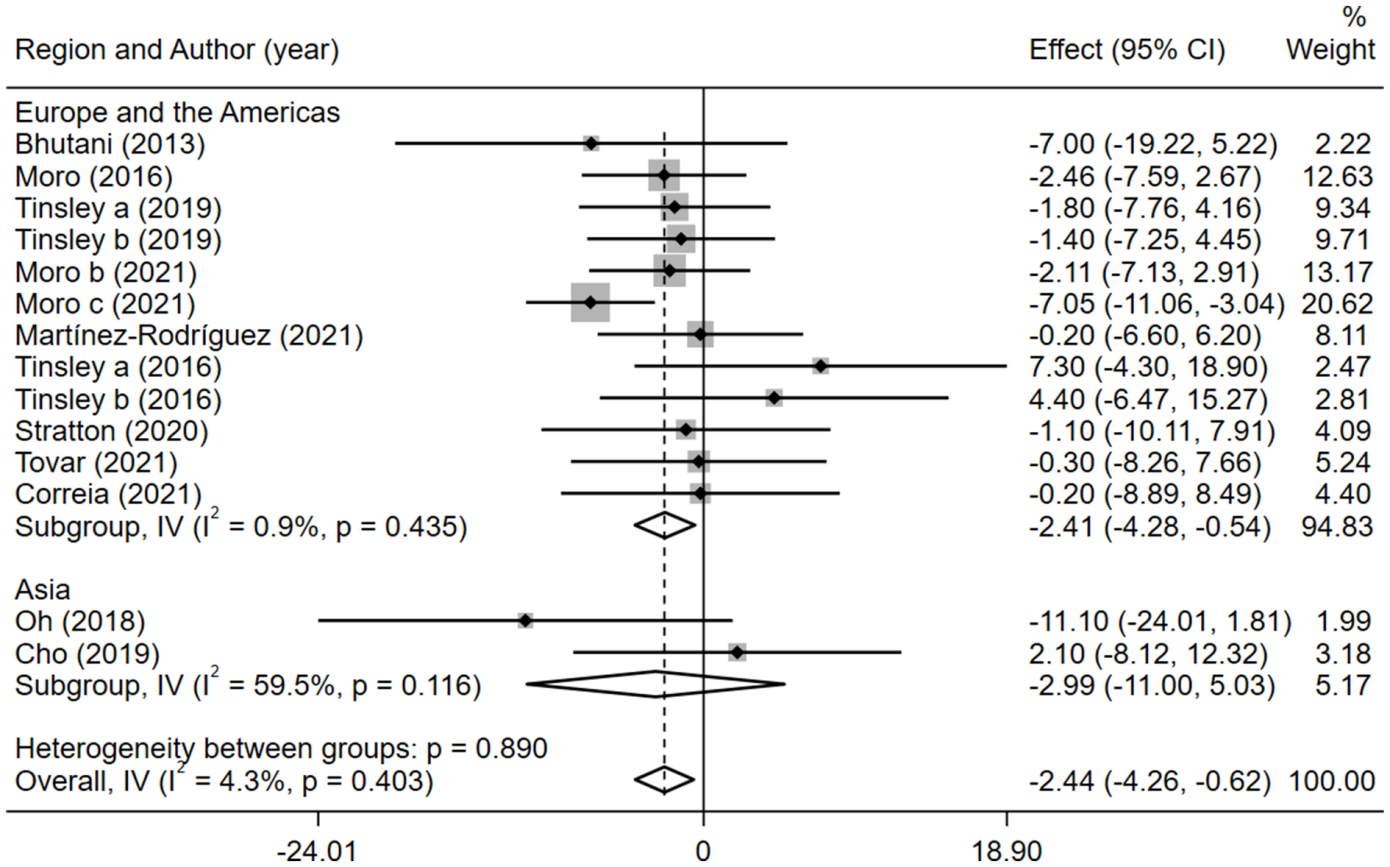
Forest plot of subgroup analysis for the effects on BM with different study regions. Each study-specific estimate is represented by a small solid diamond with adjoining horizontal lines which represent the 95% confidence intervals. The size of the gray square surrounding the study-specific estimates represents the weight of each study in the meta-analysis. The diamond with an ascending dashed line from its upper point is the summary estimate. The width of diamond represents the 95% confidence intervals of the summary estimate.

Given the effects of the intervention duration, article publication time, IF subtypes, study regions, and subject types, a subgroup analysis was performed accordingly. When the intervention duration was greater than or equal to 8 weeks, the combined intervention was significantly better than MVPA alone in reducing BM (WMD = −3.20, 95% CI −5.31 to −1.09, *p* = 0.003). However, when the intervention time was less than 8 weeks, there was no significant difference between the two intervention approaches (WMD = −0.23, 95% CI −3.83 to 3.83, *p* = 0.903; Figure 3). This suggests that the intervention duration plays a role in reducing BM in the combined intervention approach. Interestingly, according to the literature published since 2019, combined interventions have been shown to reduce BM more effectively (WMD = −2.64, 95% CI −4.71 to −0.57, *p* = 0.012); however, the difference was not significant in papers published in 2018 and before (WMD = −1.74, 95% CI −5.61 to 2.14, *p* = 0.380; Figure 4). We assume that this may be due to the increasing interest in IF in recent years and the refinement of related concepts and experiments, which may have contributed to these results. Moreover, we also found that the “TRF + MVPA” intervention indicated a better effect than MVPA alone in reducing BM levels (WMD = −2.30, 95% CI −4.26 to −0.41, *p* = 0.017), whereas no significant difference was found between the “ADF + MVPA” and the MVPA groups (Figure 5). Furthermore, a better effect in reducing BM was found in studies conducted in Europe and the Americas (WMD = −2.41, 95% CI −4.28 to −0.54, *p* = 0.012). However, BM changes in the two groups were not significantly different in Asia (Korea; WMD = −3.00, 95% CI −11.00, 5.03, *p* = 0.465; Figure 6). Finally, the effect of the combined intervention differed insignificantly from the MVPA intervention in the obese population (WMD = −4.20, 95% CI −10.90, 2.51, *p* = 0.220), whereas in the healthy population, the combined intervention showed a greater effect of changes on BM (WMD = −2.30, 95% CI −4.19, −0.41, *p* = 0.009; Supplementary Figure 5).

### 3.5. Effect on fasting blood glucose

**Figure 7:**
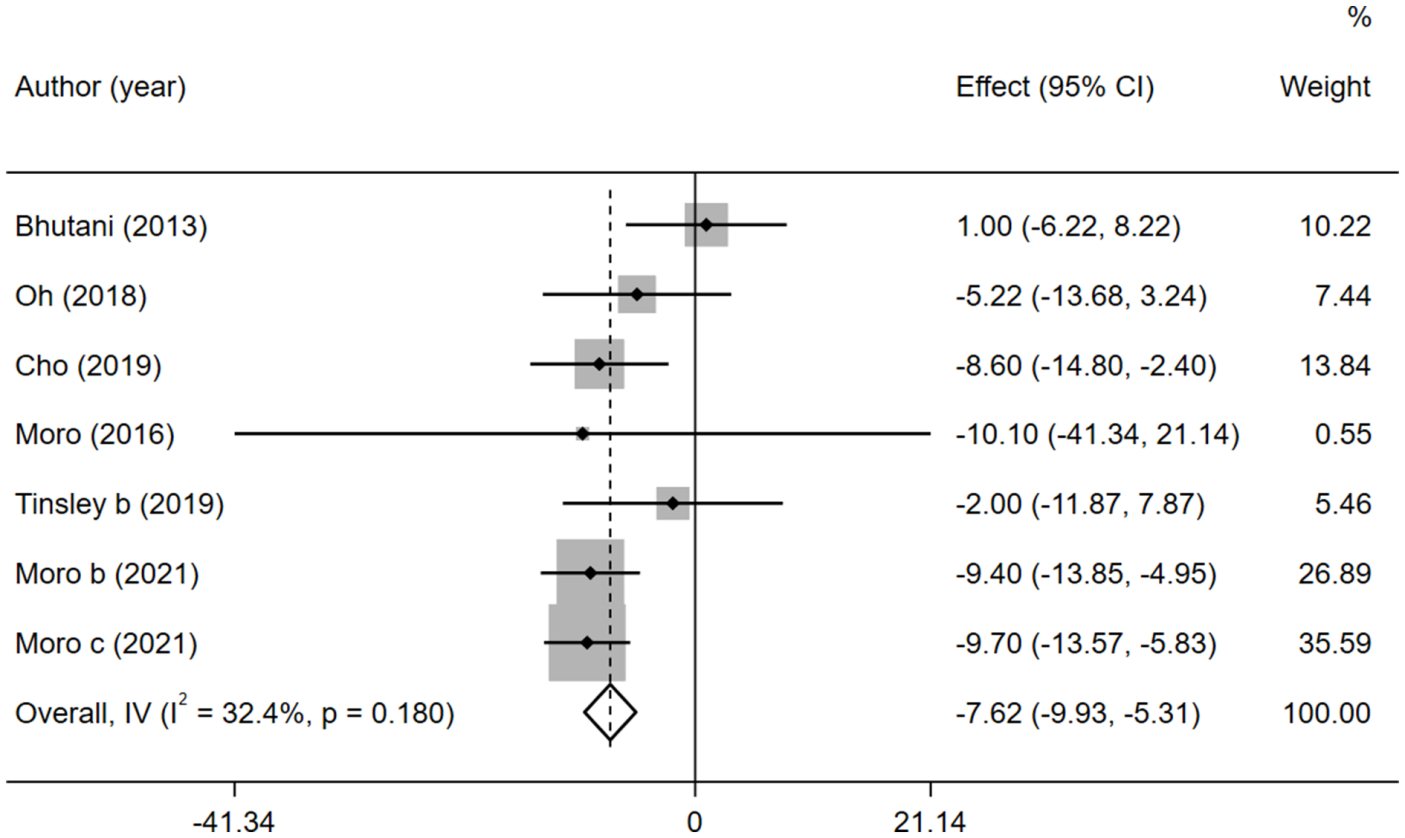
Forest plots of the overall meta-analysis for the effects on FBG levels. Each study-specific estimate is represented by a small solid diamond with adjoining horizontal lines which represent the 95% confidence intervals. The size of the gray square surrounding the study-specific estimates represents the weight of each study in the meta-analysis. The diamond with an ascending dashed line from its upper point is the summary estimate. The width of diamond represents the 95% confidence intervals of the summary estimate.

Fasting blood glucose, which reflects the function of pancreatic islet B cells (41), is also an important risk factor for MetS. Six of the 11 studies reported FBG. As a result, the combined intervention showed a significantly higher decrease in the FBG level than MVPA alone (WMD = −7.62, 95% CI −9.93 to −5.31, *p* = 0.000; Figure 7). We further performed a sensitivity analysis (Supplementary Figure 6) and the results showed that this meta-analysis was stable. No publication bias was detected using Egger’s test (*p* = 0.249; Supplementary Figure 7).

**Figure 8:**
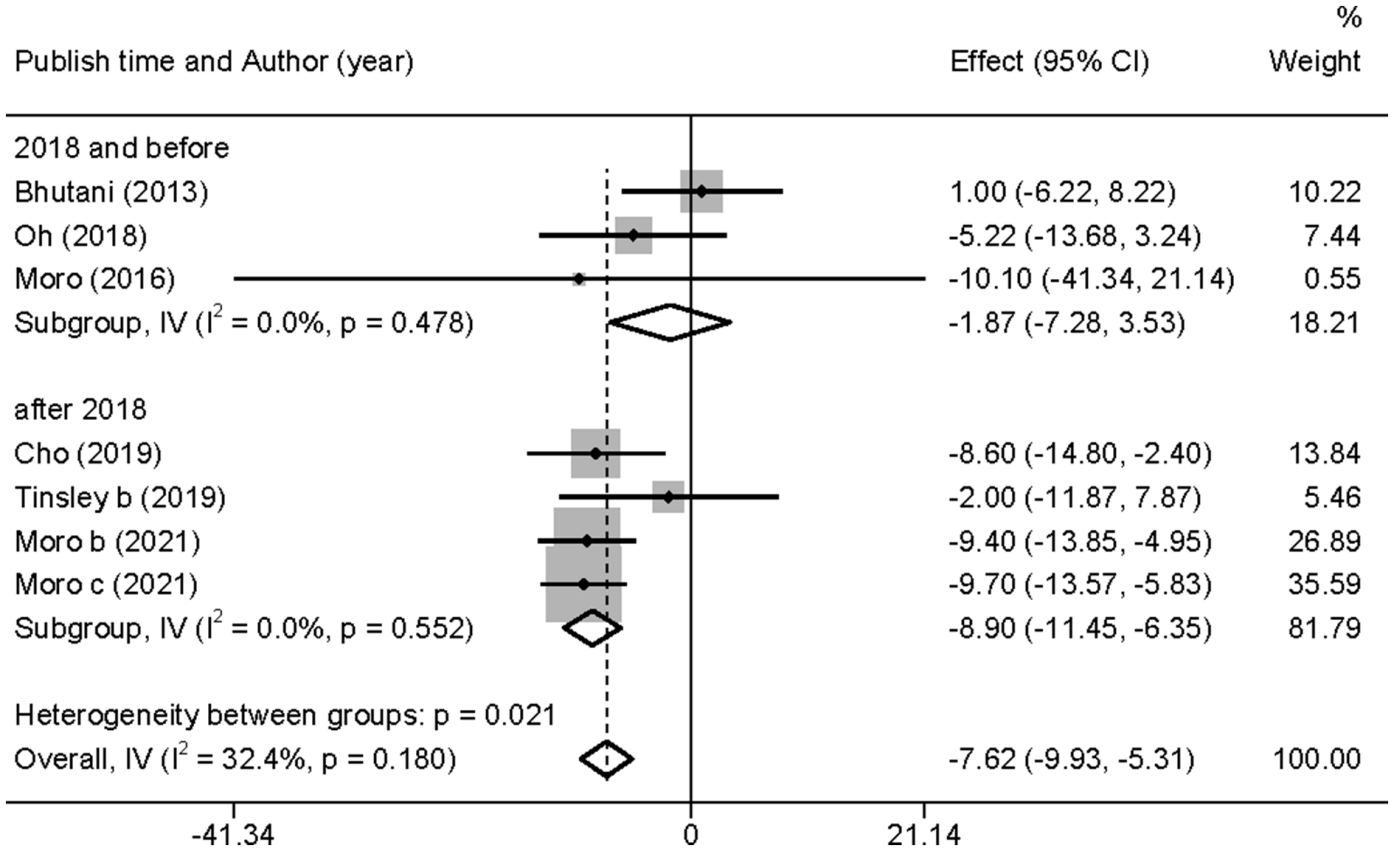
Forest plot of subgroup analysis for the effects on FBG in groups with different publication time. Each study-specific estimate is represented by a small solid diamond with adjoining horizontal lines which represent the 95% confidence intervals. The size of the gray square surrounding the study-specific estimates represents the weight of each study in the meta-analysis. The diamond with an ascending dashed line from its upper point is the summary estimate. The width of diamond represents the 95% confidence intervals of the summary estimate.

Similarly, after the subgroup analysis, we found that both ADF (WMD = −4.69, 95% CI −8.80 to 0.58, *p* = 0.025) and TRF (WMD = −8.97, 95% CI −11.76 to −6.18, *p* = 0.000) combined with MVPA intervention effectively reduced FBG levels than MVPA alone (Supplementary Figure 8). Nevertheless, no significant differences were found in the FBG levels between the combined intervention and MVPA intervention groups (WMD = −1.87, 95% CI −7.28 to 3.53, *p* = 0.497), whereas in studies published in the last 3 years (after 2018), the combined intervention group was more effective for BM reduction (WMD = −8.90, 95% CI −11.45 to −6.35, *p* = 0.000; Figure 8). Moreover, FBG levels were more significantly decreased with the combined intervention in both the European and American studies (WMD = −7.68, 95% CI −10.28 to −5.08, *p* = 0.000) and in the Asian (Korean) study (WMD = −7.42, 95% CI −12.42 to −2.42, *p* = 0.004). Similarly, the combined intervention showed greater improvement in FBG in both obese (WMD = −4.69, 95% CI −8.80 to −0.58, *p* = 0.025) and healthy (WMD = −8.97, 95% CI −11.76 to −6.18, *p* = 0.000) population subgroups (Supplementary Figure 9).

### 3.6. Effect on lipid metabolism indicators

Studies have shown that lipid metabolism disorders are the key causative factors for MetS (42). In this study, we re-analyzed TG and HDL-C levels to evaluate the effects of different interventions.

**Figure 9:**
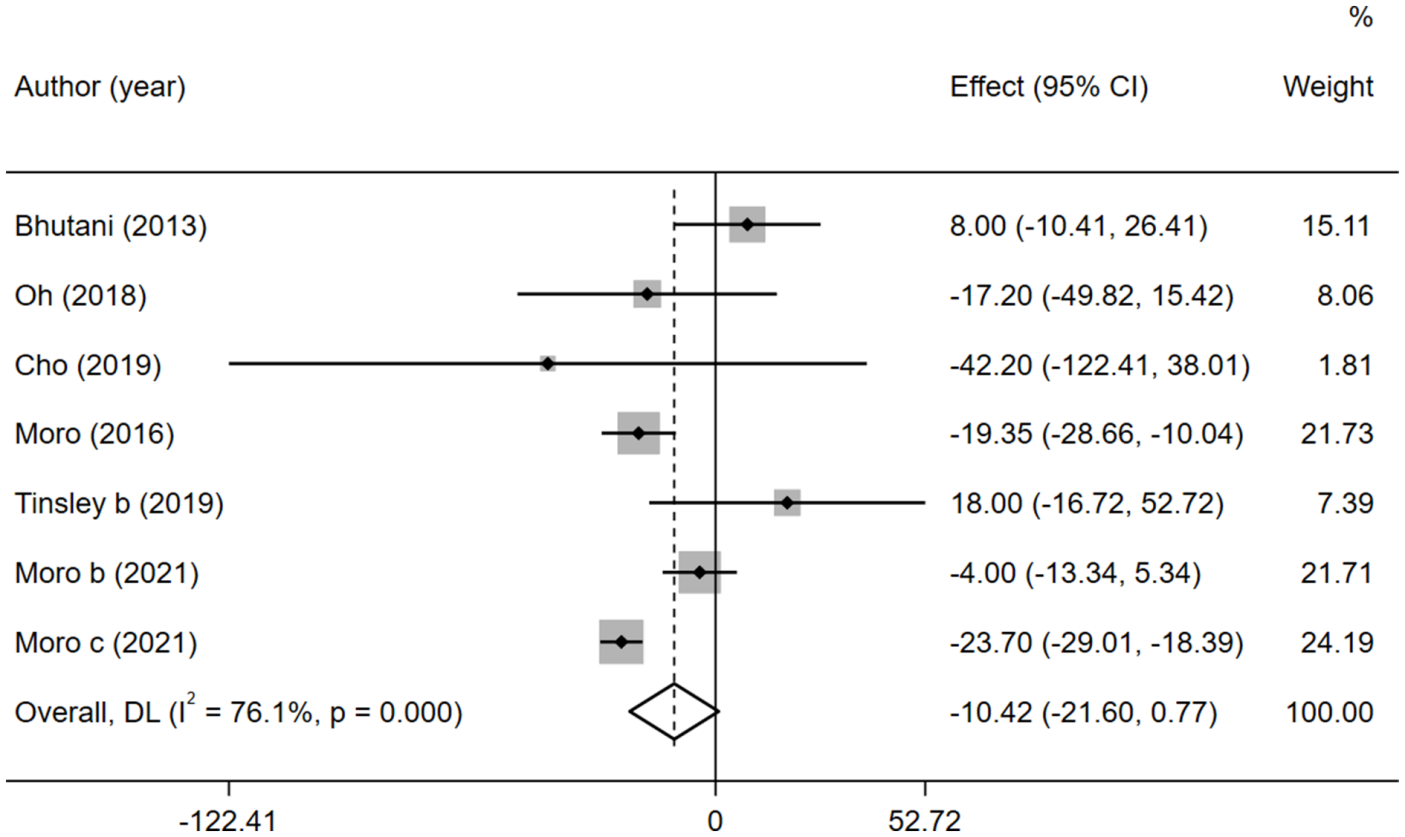
Forest plot of the overall meta-analysis for the effects on TG levels. Each study-specific estimate is represented by a small solid diamond with adjoining horizontal lines which represent the 95% confidence intervals. The size of the gray square surrounding the study-specific estimates represents the weight of each study in the meta-analysis. The diamond with an ascending dashed line from its upper point is the summary estimate. The width of diamond represents the 95% confidence intervals of the summary estimate.

According to the heterogeneity test, there was significant heterogeneity among the six studies with TG ( $I^{2}$ = 76.1%, *p* < 0.05); therefore, the random-effects model was adopted. No significant difference was found between the combined intervention and MVPA intervention groups (WMD = −10.42, 95% CI −21.61 to 0.77, *p* = 0.068; Figure 9). The sensitivity analysis (Supplementary Figure 10) showed that the results were stable. Egger’s publication bias plot (Supplementary Figure 11) indicated that no publication bias existed in the studies (*p* = 0.251).

**Figure 10:**
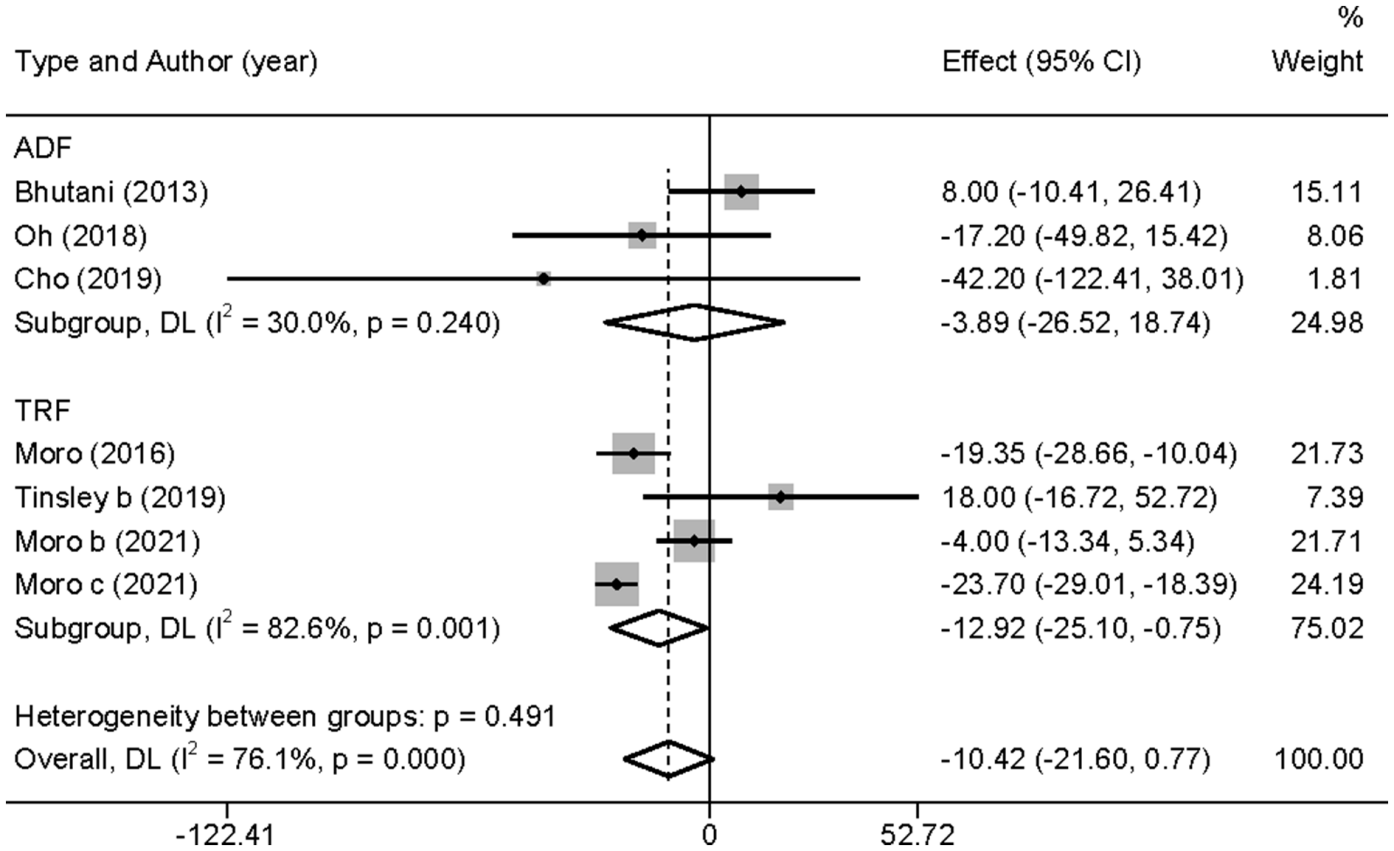
Forest plot of subgroup analysis for the effects on TG in groups with different IF subtypes. Each study-specific estimate is represented by a small solid diamond with adjoining horizontal lines which represent the 95% confidence intervals. The size of the gray square surrounding the study-specific estimates represents the weight of each study in the meta-analysis. The diamond with an ascending dashed line from its upper point is the summary estimate. The width of diamond represents the 95% confidence intervals of the summary estimate.

However, when subgroup analyzed according to IF types (TRF and ADF), we found that TRF combined with MVPA significantly decreased TG levels than MVPA alone (WMD = −12.92, 95% CI −25.10 to −0.75, *p* = 0.037), whereas ADF combined with MVPA intervention did not (WMD = −3.89, 95% CI −26.52 to 18.74, *p* = 0.736; Figure 10). This suggests that, compared with ADF, TRF combined with MVPA is more suitable for individuals with high TG levels. Moreover, neither the subgroup analysis based on the article publication time (before 2018 [WMD = −9.46, 95% CI −29.04 to 10.15, *p* = 0.344] and after 2018 [WMD = −10.15, 95% CI −28.20 to 7.89, *p* = 0.270]) nor that based on study regions (European and American studies [WMD = −8.89, 95% CI −21.25 to 3.48, *p* = 0.159] and Korean study [WMD = −20.75, 95% CI −50.96 to 9.47, *p* = 0.178]) were significantly different (Supplementary Figures 12, 13). However, the combined intervention was not more effective than the MVPA intervention in the obese population (WMD = −3.89, 95% CI −26.52 to 18.74, *p* = 0.736), but the combined intervention showed more significant changes in TG levels in the healthy population (WMD = −12.92, 95% CI −25.10 to −0.75, *p* = 0.037; Supplementary Figure 14).

Similarly, six studies reported the involvement of HDL-C. Concerning the low heterogeneity ( $I^{2}$ = 49.8%, *p* > 0.05), a fixed-effects model was applied. Furthermore, a sensitivity analysis (Supplementary Figure 15) revealed that the 2021 study by Moro et al. (35) was a source of heterogeneity. And no evidence of publication bias was found by the Egger’s test (*p* = 0.794; Supplementary Figure 16).

**Figure 11:**
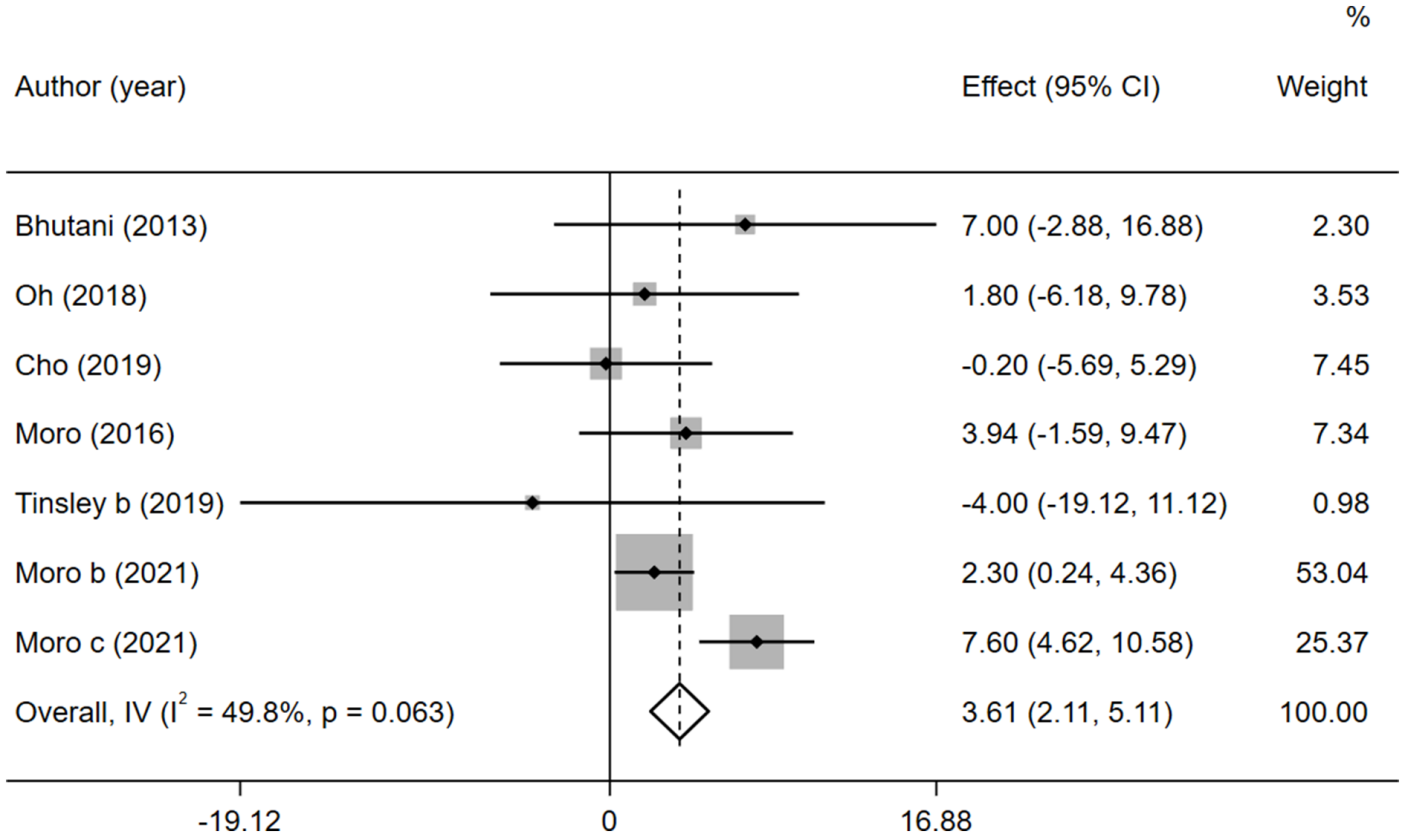
Forest plot of the overall meta-analysis for the effects on HDL-C levels. Each study-specific estimate is represented by a small solid diamond with adjoining horizontal lines which represent the 95% confidence intervals. The size of the gray square surrounding the study-specific estimates represents the weight of each study in the meta-analysis. The diamond with an ascending dashed line from its upper point is the summary estimate. The width of diamond represents the 95% confidence intervals of the summary estimate.

**Figure 12:**
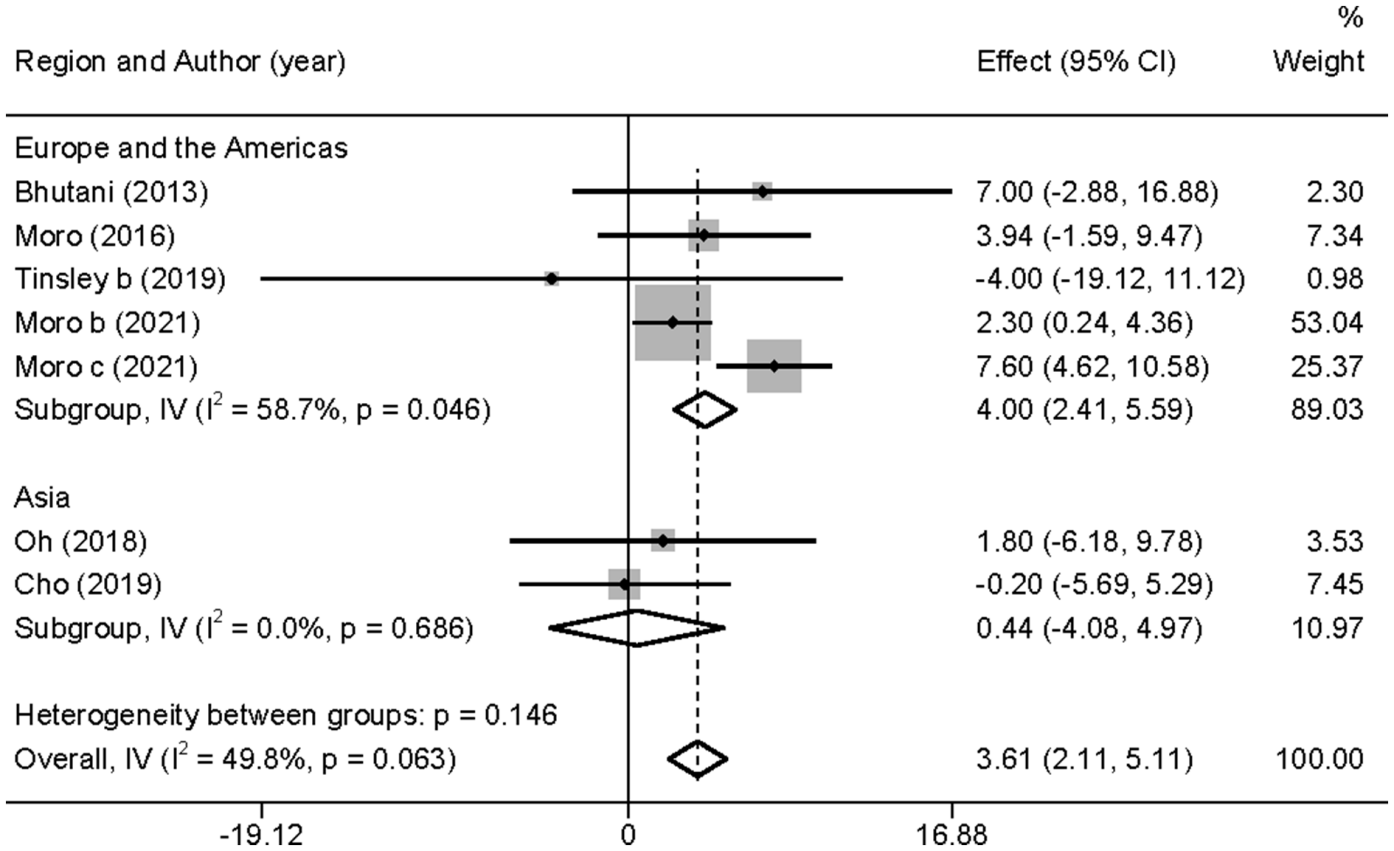
Forest plot of subgroup analysis for the effects on HDL-C in groups with different study regions. Each study-specific estimate is represented by a small solid diamond with adjoining horizontal lines which represent the 95% confidence intervals. The size of the gray square surrounding the study-specific estimates represents the weight of each study in the meta-analysis. The diamond with an ascending dashed line from its upper point is the summary estimate. The width of diamond represents the 95% confidence intervals of the summary estimate.

In contrast to TG level, we found that the combined intervention significantly better increased HDL-C levels than MVPA alone (WMD = 3.61, 95% CI 2.11–5.11, *p* = 0.000; Figure 11). Subgroup analysis of IF subtypes showed that TRF combined with MVPA (WMD = 3.92, 95%CI 2.31–5.53, *p* = 0.000) was a favorable factor for HDL-C elevation compared with ADF combined with MVPA (WMD = 1.58, 95% CI −2.54 to 5.69, *p* = 0.452; Supplementary Figure 17). This was similar for TG, suggesting that TRF combined with MVPA should be highly recommended. Moreover, a significant increase in HDL-C level by the combined intervention was observed in studies published in the last 3 years (after 2018; WMD = 3.56, 95% CI 1.95 to 5.17, *p* = 0.000), whereas no better effect on improving HDL-C levels was observed in studies published before 2018 (WMD = 3.90, 95% CI −0.23 to 8.03, *p* = 0.064; Supplementary Figure 18). Notably, the combined intervention showed a significant increase in HDL-C levels than MVPA alone in European and American studies (WMD = 4.00, 95% CI 2.41 to 5.58, *p* = 0.000). However, studies in Asia (Korea) showed no significant difference in the HDL-C level (WMD = 0.44, 95% CI −4.08 to 4.97, *p* = 0.848; Figure 12). This suggests that the combination intervention may be more suitable for Caucasians with low HDL-C levels. In the subgroup analysis based on different populations, we found that in the obese population, the difference between the combined intervention and the MVPA intervention effect was not significant (WMD = 1.58, 95% CI −2.54 to 5.69, *p* = 0.452), while in the healthy population, the combined intervention was more effective in improving HDL-C (WMD = 3.92, 95%CI 2.31–5.53, *p* = 0.000; Supplementary Figure 19).

### 3.7. Effect on blood pressure

**Figure 13:**
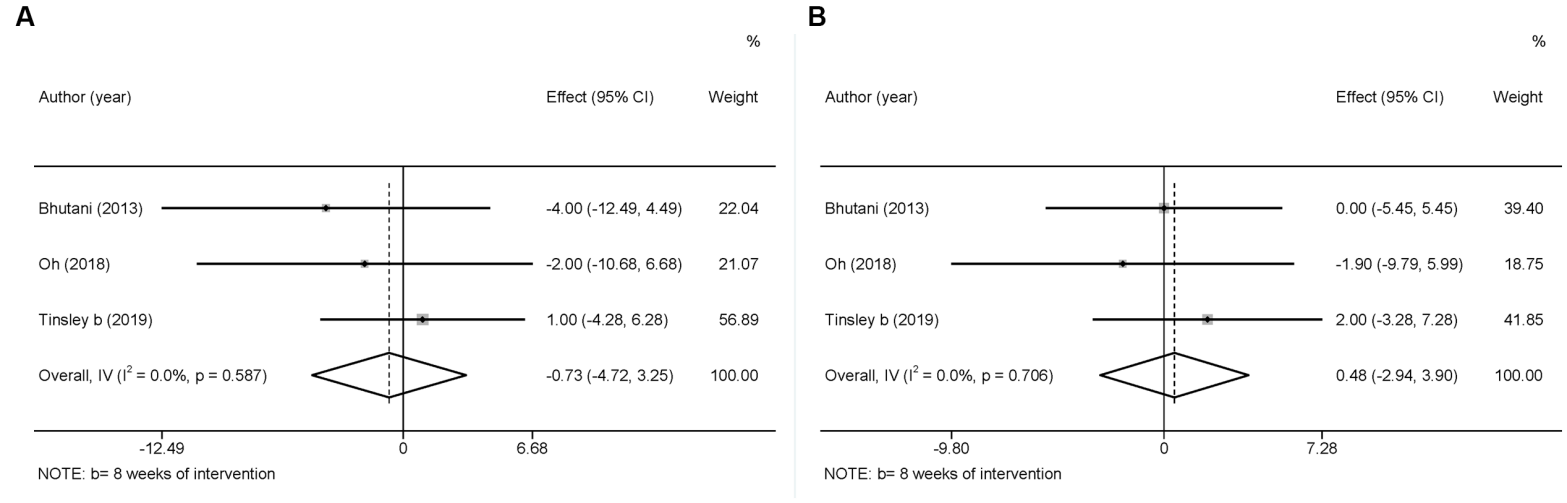
Forest plots of the meta-analysis for the effects on blood pressure. **(A)** SBP levels, **(B)** DBP levels. Each study-specific estimate is represented by a small solid diamond with adjoining horizontal lines which represent the 95% confidence intervals. The size of the gray square surrounding the study-specific estimates represents the weight of each study in the meta-analysis. The diamond with an ascending dashed line from its upper point is the summary estimate. The width of diamond represents the 95% confidence intervals of the summary estimate.

Blood pressure (BP), which is used to determine cardiac function and peripheral vascular resistance (43), is also a potential risk factor for MetS (44). Three studies used SBP and DBP as outcome variables. In view of the low heterogeneity in SBP ( $I^{2}$ = 0.0%, *p* > 0.05) and DBP ( $I^{2}$ = 0.0%, *p* > 0.05), a fixed-effects model was employed. The results showed a slight decreasing tendency in SBP (WMD = −0.73, 95%CI −4.72 to 3.25, *p* = 0.718) and a slight increasing tendency in DBP (WMD = 0.48, 95%CI −2.94 to 3.90, *p* = 0.783), However, these changes were not statistically significant (Figure 13).

Moreover, there was no significant difference between the intervention and control groups in terms of changes in SBP and DBP in the original studies.

### 3.8. Combination group compared with IF group

Because IF alone could lead to nutritional deficiencies (45), few studies have focused on IF alone for intervention in MetS. In this study, we gathered data from three multi-arm randomized controlled trial studies evaluating the effects of MVPA-IF combination vs. IF alone on weight loss and cardiometabolic risk factors. However, probably due to the limited amount of data, all meta-analysis results on BM, FBG, TG, HDL-C, SBP, and DBP were not statistically significant (Supplementary Table 4).

## 4. Discussion

This study aimed to investigate effective approaches to improve the risk factors for MetS. In summary, our meta-analysis demonstrated the following four points: (a) Combining IF with MVPA was more effective than MVPA alone in improving BM, FBG, and HDL-C levels in adults, especially when MVPA was combined with TRF and the duration of the intervention was more than 8 weeks. Combined interventions may be more effective in preventing and managing MetS than MVPA alone; (b) The combined intervention did not show significant changes in the TG level and BP, although stronger effects were observed on the TG level in the TRF combined with MVPA subgroup; (c) More significant improvements in BM, FBG, and HDL-C levels were found in the subgroup of studies published from 2019 to 2021 (the last 3 years); (d) Compared with Asian studies, studies conducted in Europe and the Americas reported significant improvements in BM, FBG levels, and HDL-C levels by the combined intervention. (e) In the obese population, the combined intervention only had a more significant effect on FBG, while in the healthy population, the combined intervention showed more beneficial changes in BM, FBG, TG, and HDL-C. Overall, combining IF with MVPA was effective in reducing BM and FBG levels while significantly elevating HDL-C levels, suggesting that IF combined with MVPA may be better for preventing and treating individuals with these risk factors. In contrast, for patients with abnormal TG and BP levels, the combined intervention was not as effective, indicating that it may not be suitable for those with these risk factors.

In our main outcomes, IF combined with MVPA significantly increased HDL-C levels (*p* = 0.000) but failed to significantly reduce TG levels simultaneously (*p* = 0.068). Although a decrease in TG implies a concomitant increase in HDL-C in general, our results, which did not show this trend, may be due to the potentially complex relationship between HDL-C and TG in patients with MetS, which is similar to that in patients with other diseases (46). To gain a deeper insight, we performed a subgroup analysis based on IF subtypes. The results showed that ADF combined with MVPA did not significantly improve both HDL-C and TG, although TRF combined with MVPA significantly improved both HDL-C and TG levels. This may be because TRF improves circadian rhythms (47) or alters biological clock gene expression directly (48). Additionally, ADF has been reported to have little effect on improving glycemic control and metabolism-related body composition (23, 49). The investigators also speculated that the negative results may be due to poor compliance with ADF, as the participants frequently consumed foods outside the prescribed diet (23).

Studies on combined interventions have shown opposite results in different regions and populations. Combined interventions showed better results in studies conducted in Europe and the Americas, whereas no significant differences were found in studies conducted in Asia. Coincidentally, the subjects in the study conducted in Europe and the United States included healthy trained people, while the study conducted in Asia included obese people. The different subject types may be an important factor in this result. Similarly, subgroup analyses based on subject types showed that the combined intervention was more effective in the healthy population, whereas in the obese population, the combined intervention did not show a better effect than the single intervention. In the studies of the obese population, the single intervention already made a significant improvement on MetS risk factors (30–32), so the combined interventions may not be a better option. In contrast, combined interventions are more meaningful for the prevention of MetS risk factors in the healthy population. However, we speculate that this may also be due to the small number of studies collected on Asian populations (31, 32). Therefore, further RCT studies with larger sample sizes are required to strengthen this conclusion.

Subgroup analysis at the time of publication showed that the combined intervention significantly improved BM, FBG level, and HDL-C level than MVPA alone in the context of social development and dietary changes. Over the past decade, IF has emerged as a more effective alternative to traditional CR, potentially reducing body weight and is recommended for improving metabolic health (16, 50). Meanwhile, there is an increase in the public’s awareness of chronic disease and lifestyle improvements, including diet and physical activity (51, 52). These changes may have improved patient compliance, which may explain why the combined interventions showed better results in studies conducted after 2018. Moreover, in recent years, intervention programs combining IF with PA have been proposed more frequently, which may also be one of the reasons for the more significant results (53–55).

Due to limited number of involved studies, we only explored the influence of intervention duration on the effect on BM, and the results showed a more significant effect when the intervention lasted 8 weeks or more. This may be related to the long-term mechanism of IF and MVPA in MetS (56). Long-term exercise clearly benefits metabolic health (57). Beige fat is known to be beneficial in improving metabolic diseases (58). Li et al. (16) showed that ADF may play a key role in inducing beige fat production, and this effect becomes more pronounced with time. Similarly, Chaix et al. identified that TRF improved the biological clock and metabolism by driving the shift from glucose to lipid metabolism, and this effect increased in a time-dependent manner (56). As mentioned above, the intervention duration is a variable of interest. In our study, the duration of the intervention reached a maximum of 48 weeks, whereas in all other studies, it was between 4 and 12 weeks. This may be the main reason for the heterogeneity observed in the results.

We found that subjects in all three studies with BP as an outcome indicator had normal baseline BP. Comparing to blank control, the combined intervention and the single intervention did not cause significant changes in BP (30, 31, 34). Our meta-analysis suggests that the combination of IF with MVPA does not have a greater effect on BP changes. Based on previous studies, every single intervention is effective for people with elevated BP (59–62). Therefore, we presume that the combined intervention failed to significantly improve BP mainly due to the normal baseline level. When future combined interventions of IF and MVPA are implemented in a hypertensive population, we may be able to draw further conclusions about the effect of the combined intervention on BP normalization. In addition, the limited number of participants included in this study may also explain why no differences were observed. Therefore, more research data is required to obtain a clear conclusion.

Furthermore, our study showed that the combined intervention was not more effective than IF alone in improving any metabolic risk factor. Interestingly, previous studies have reported that IF alone was more effective in reducing BM than a combined intervention (55). Thus, IF, with or without MVPA intervention, is an effective way to improve metabolic risk factors, but the comparison of effectiveness between them needs to be further investigated and validated.

This study had several limitations. First, the sample size in this study was relatively small because of our strict inclusion criteria as well as the fact that there are currently few studies on combined interventions. Similarly, inadequate sample size leads to another substantial limitation, namely the heterogeneity of the study sample. Although we performed subgroup analyses of obese and healthy populations to reduce heterogeneity, there were still differences in body composition and physical activity at baseline in the included samples. The larger the sample size, the stronger the statistical power. More research data are expected to enhance the reliability of conclusions. Second, we compared the effects of the combined intervention with MVPA or IF alone, but our study lacked the comparison of effects between MVPA and IF. Future studies could consider using a network meta-analysis to compare the effects of these three interventions. Third, owing to the limitations of the original literature, BM was selected as an indicator for evaluating the effect of weight loss. In the future, more consideration should be given to body mass index and waist circumference to evaluate the effectiveness of interventions (63–65). Finally, only three interventions in our collection lasted 12 weeks or longer. Most studies lasted between 4 and 8 weeks; therefore, our analysis could not determine long-term changes in the MetS risk factors.

## 5. Conclusion

This study showed that different interventions had different effects on the various risk factors for MetS. Combined intervention with IF and MVPA, especially TRF combined with MVPA, was more beneficial than MVPA alone for individuals with increased FBG, TG, and HDL-C levels, and/or BM. However, the combined intervention had no significant effect on BP changes compared with IF or MVPA alone. Thus, different interventions may be appropriate for individuals with various risk factors. The identified combined interventions provide a new perspective on special effective interventions for MetS risk factors.

## Author contributions

HW contributed to the evaluation and interpretation of data, and the writing of the first drafts and the final version of this manuscript. YQ and BL contributed to the development of the review concept, assisted in the editing of all drafts of the manuscript, and critically reviewed the manuscript. YD contributed to the primary evaluation and interpretation of data, assisting in editing of all drafts of the manuscript and performed the statistical analysis. SH contributed to scrub data and maintaining research data for initial use and later re-use, and also performed much of the primary quality-of-evidence assessment. SR assisted in editing of all drafts of the manuscript and critically reviewed the manuscript. All authors contributed to the article and approved the submitted version.

## Funding

This research was funded by the Natural Science Foundation of Hunan Province in China, grant number: 2021JJ31070 and the Social Science Foundation Education Special Project of Hunan Province in 2021, grant number: 21YBJ24.

## Conflict of interest

The authors declare that the research was conducted in the absence of any commercial or financial relationships that could be construed as a potential conflict of interest.

## Publisher’s note

All claims expressed in this article are solely those of the authors and do not necessarily represent those of their affiliated organizations, or those of the publisher, the editors and the reviewers. Any product that may be evaluated in this article, or claim that may be made by its manufacturer, is not guaranteed or endorsed by the publisher.
