# Supplementary material for: A new perspective on special effective interventions for metabolic syndrome risk factors: a systematic review and meta-analysis
Source: Front Public Health. 2023 Jul 14;11:1133614. doi: 10.3389/fpubh.2023.1133614 (PMC10375293; doi:10.3389/fpubh.2023.1133614)
Supplement: Supplementary file 1 [file Data_Sheet_1.docx]

Supplementary Material

# Supplementary Figures and Tables

## Supplementary Figures


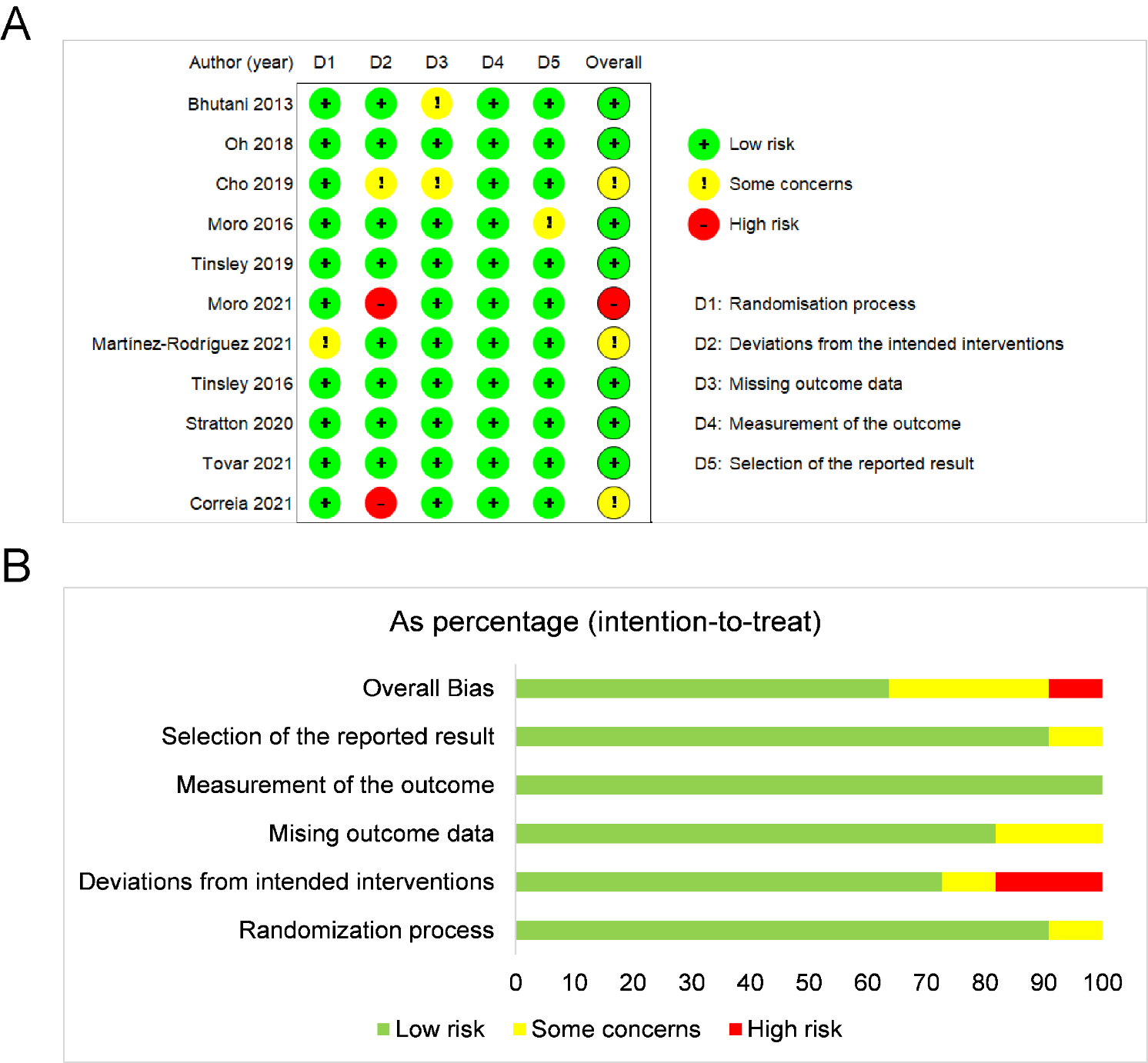


**Supplementary Figure 1.** Risk of Bias of Included Randomized Controlled Trials. (A) Risk of bias graph per type of bias assessed. “+”: low risk of bias, “-”: high risk of bias, “?”: some concerns of risk bias. (B) Risk of bias summary for the studies assessed.


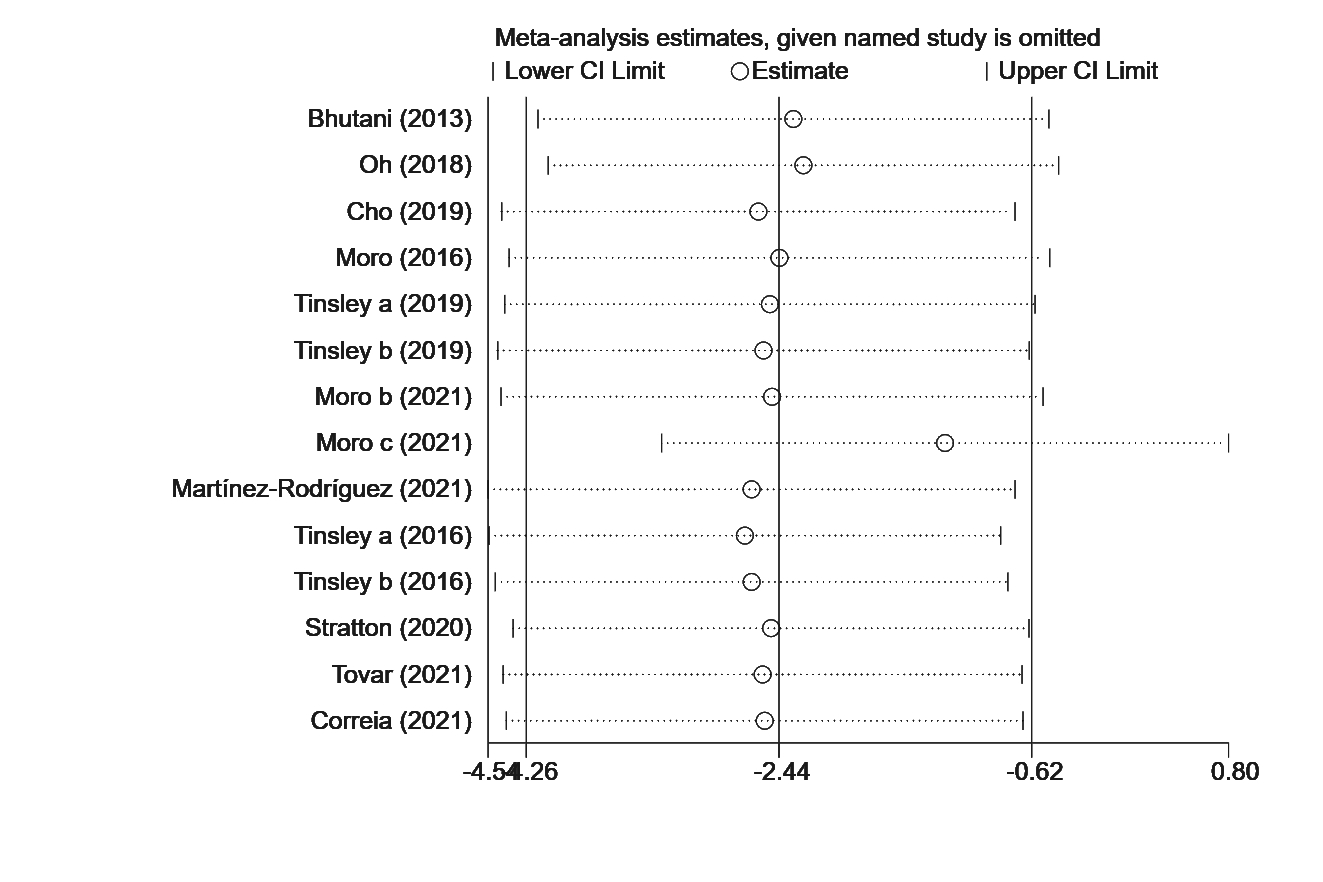


**Supplementary Figure 2.** Sensitivity analysis outcome for BM.


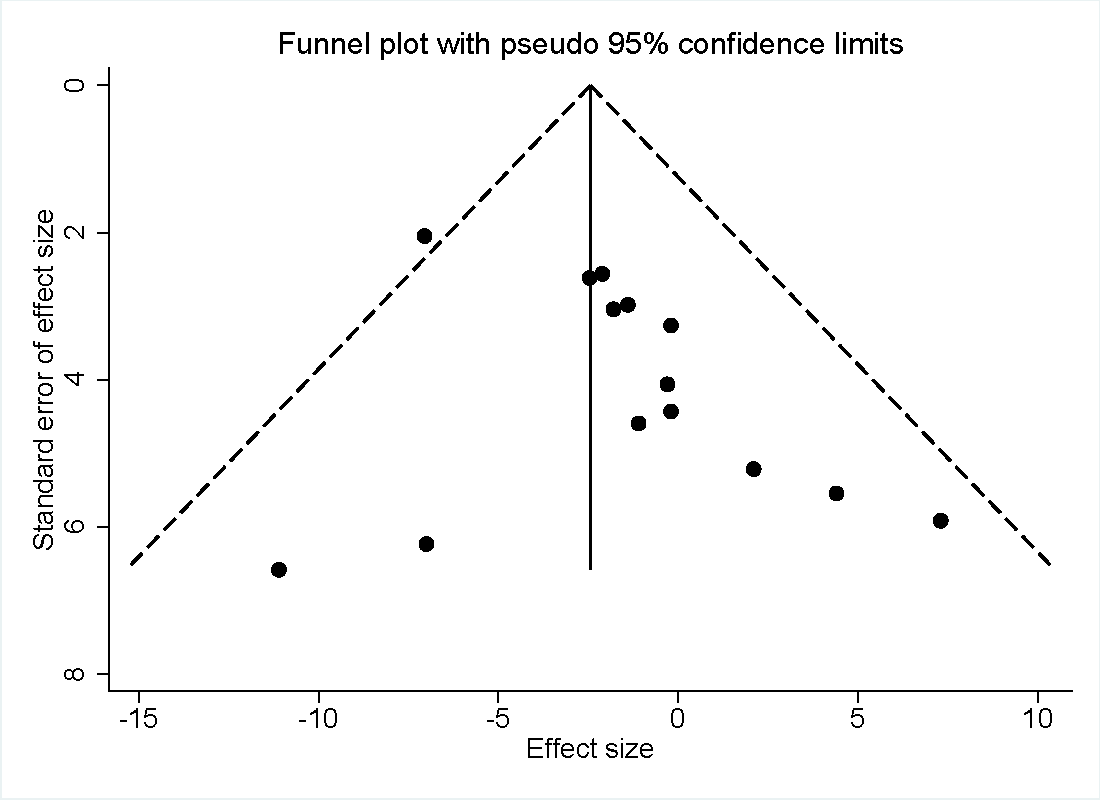


**Supplementary Figure 3.** Funnel plot for BM.


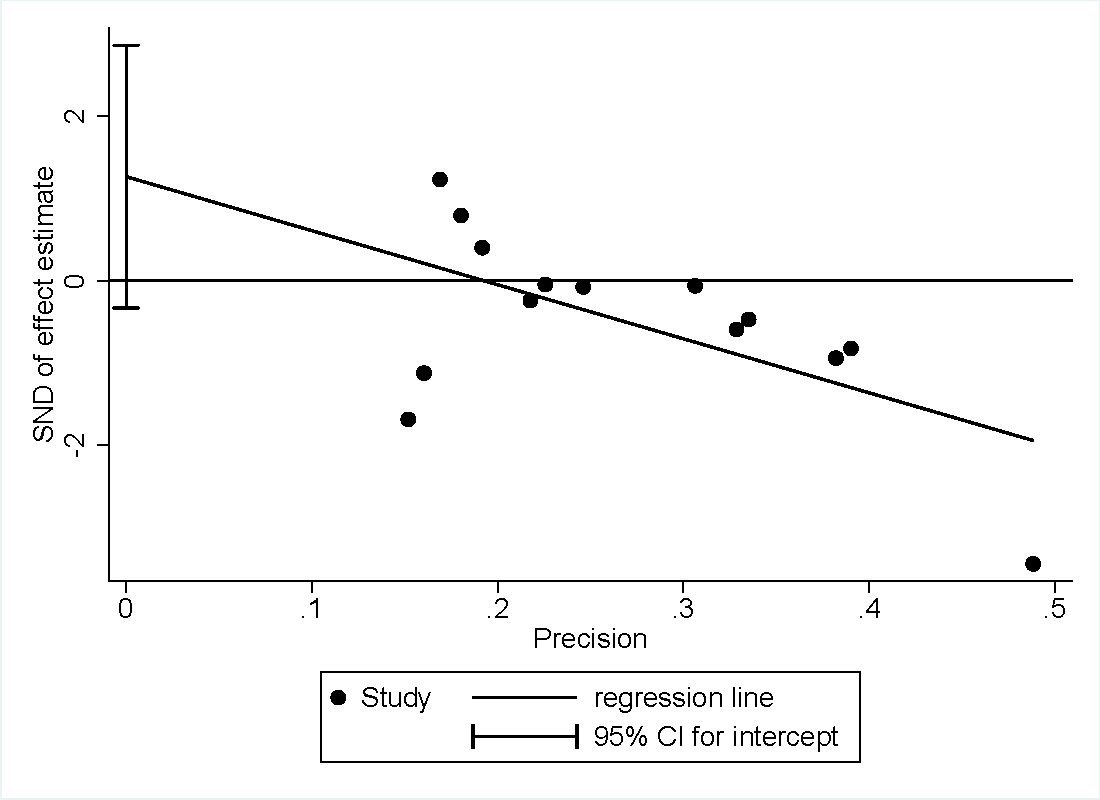


**Supplementary Figure 4.** Egger’s test plot for BM.


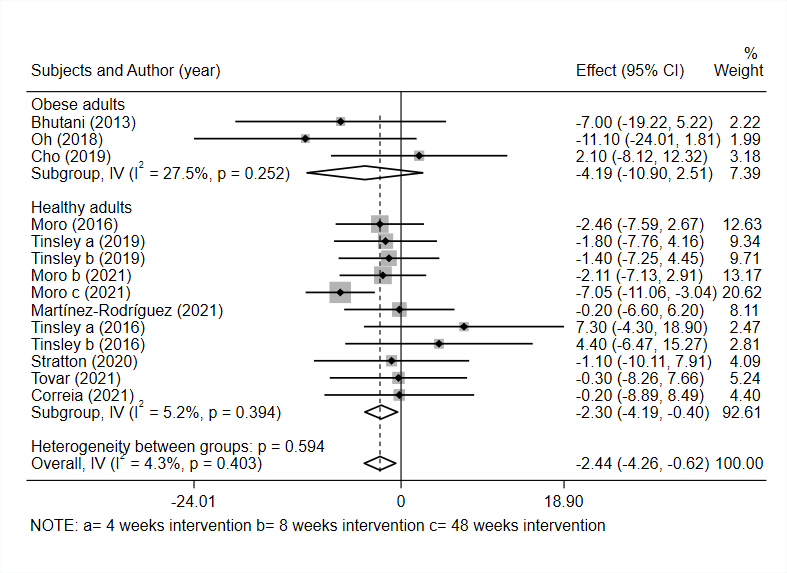


**Supplementary Figure 5.** Forest plot of subgroup analysis for the effects on BM in groups with different subject types.


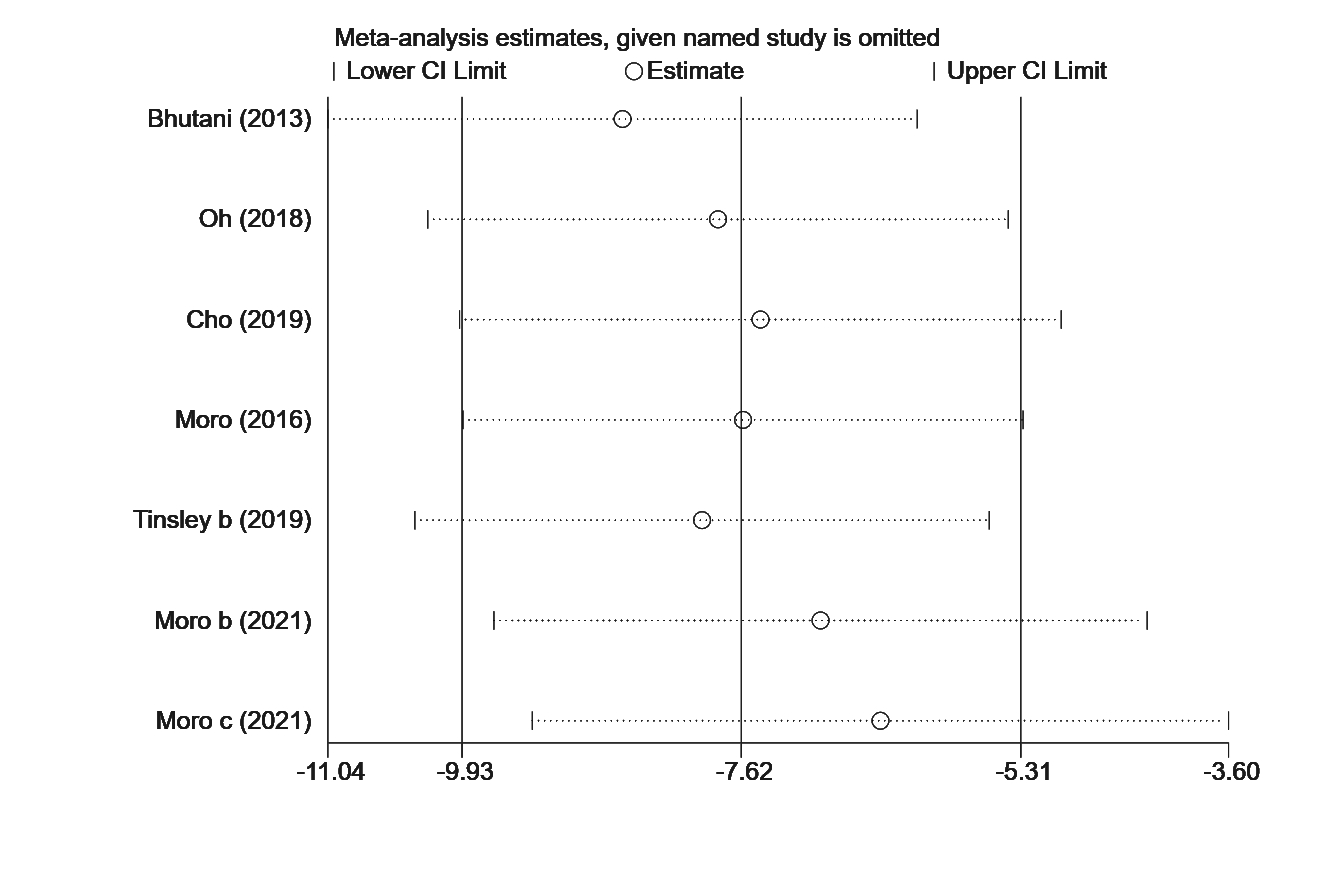


**Supplementary Figure 6.** Sensitivity analysis outcome for FBG.


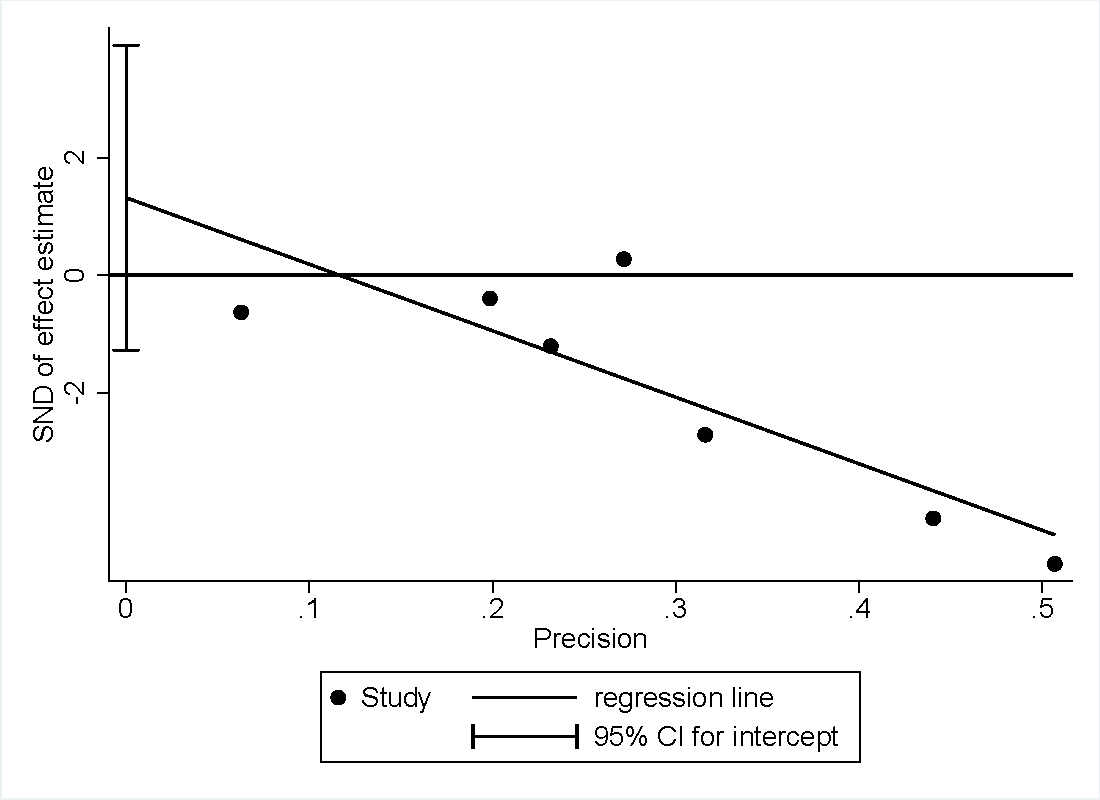


**Supplementary Figure 7.** Egger’s test plot for FBG.


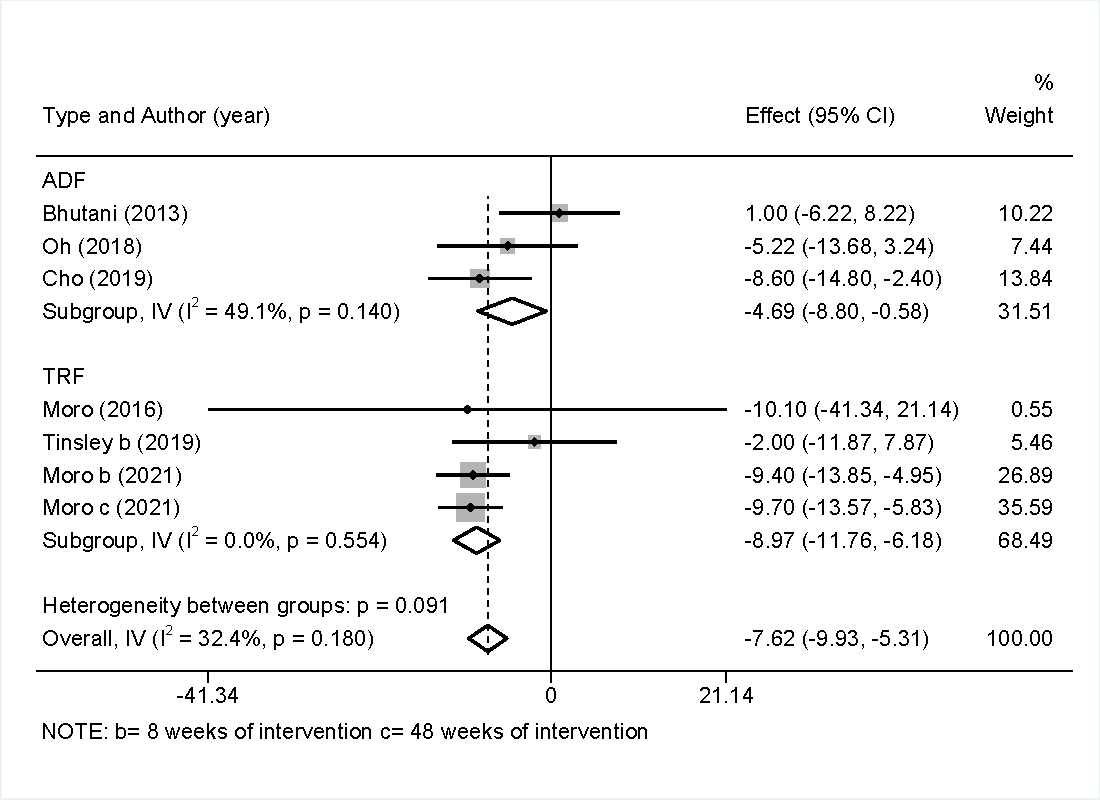


**Supplementary Figure 8.** Forest plot of subgroup analysis for the effects on FBG in groups with different IF subtypes.


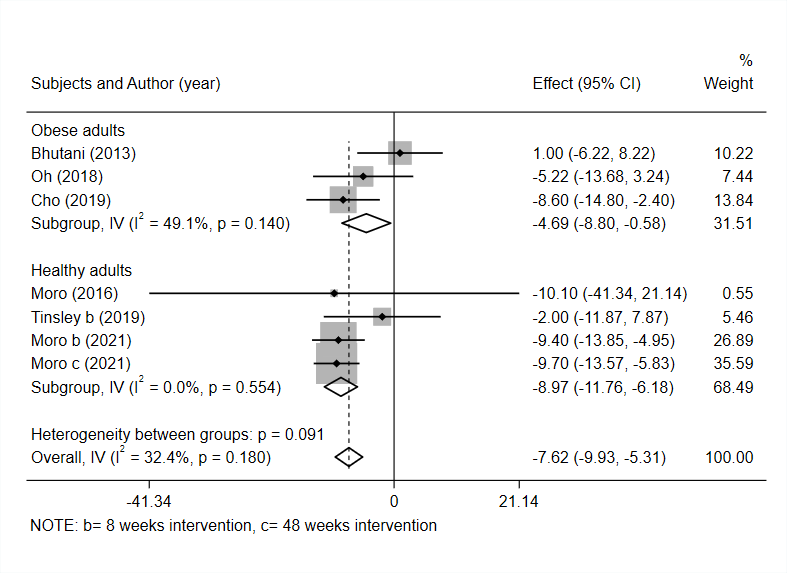


**Supplementary Figure 9.** Forest plot of subgroup analysis for the effects on FBG in groups with different subject types.


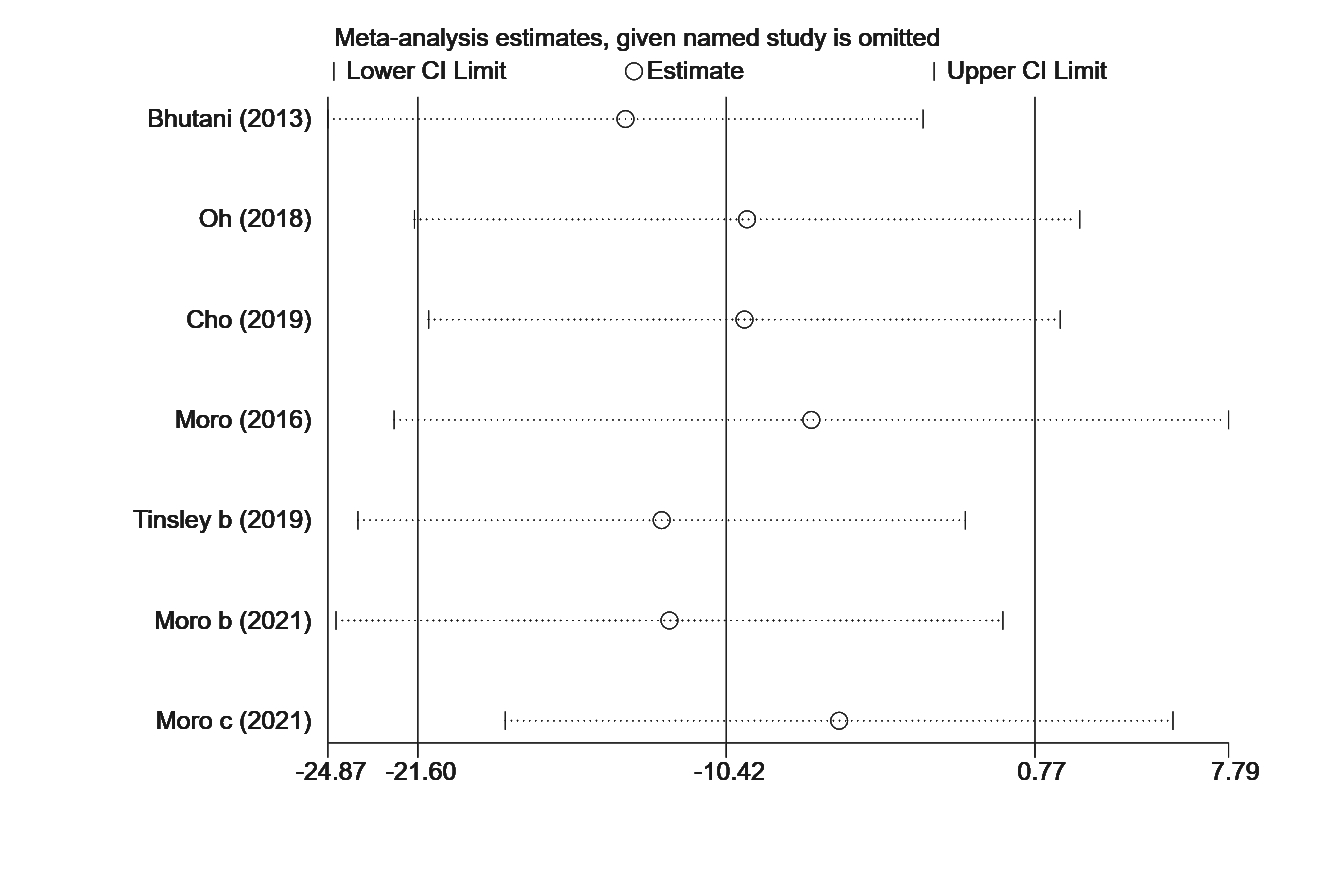


**Supplementary Figure 10.** Sensitivity analysis outcome for TG.


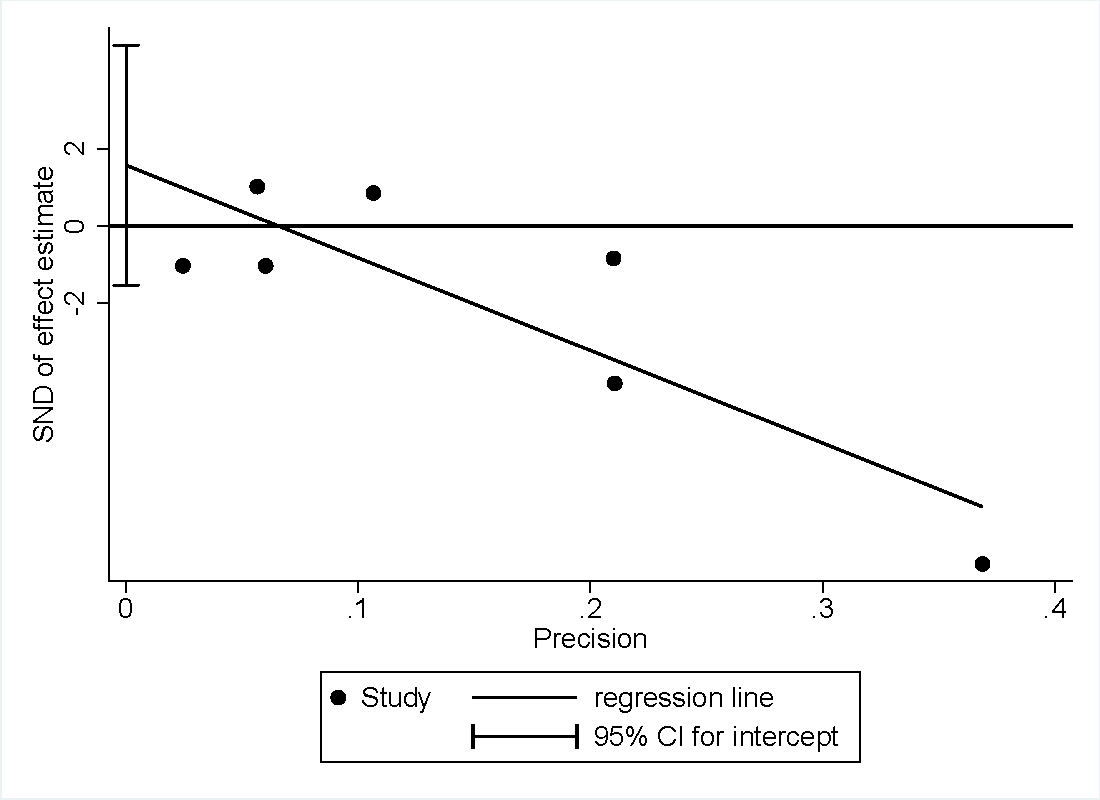


**Supplementary Figure 11.** Egger’s test plot for TG.


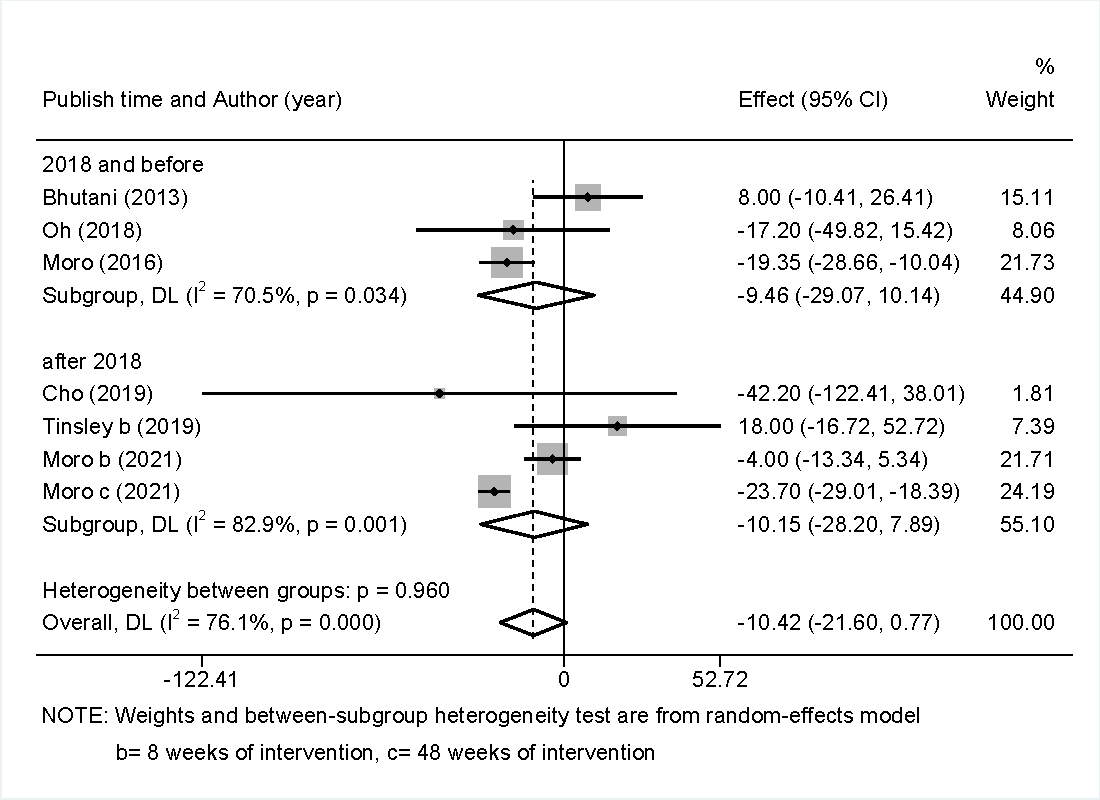


**Supplementary Figure 12.** Forest plot of subgroup analysis for the effects on TG in groups with different publication time.


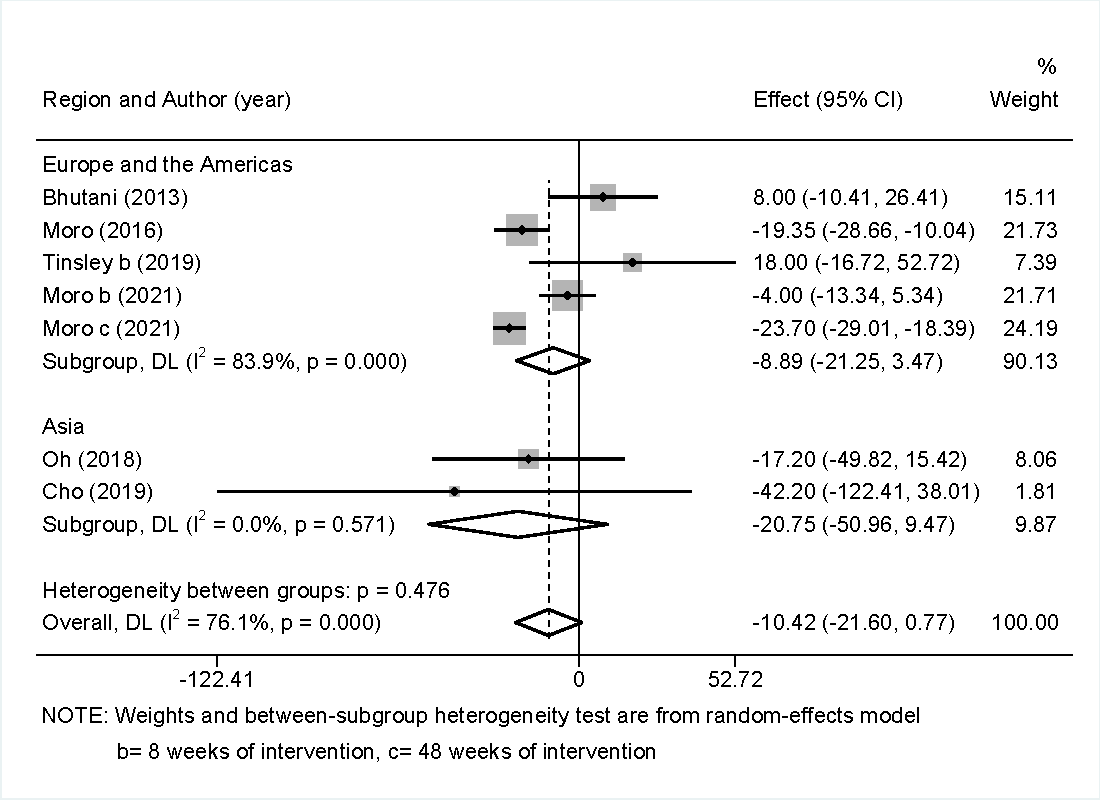


**Supplementary Figure 13.** Forest plot of subgroup analysis for the effects on TG in groups with different study regions.


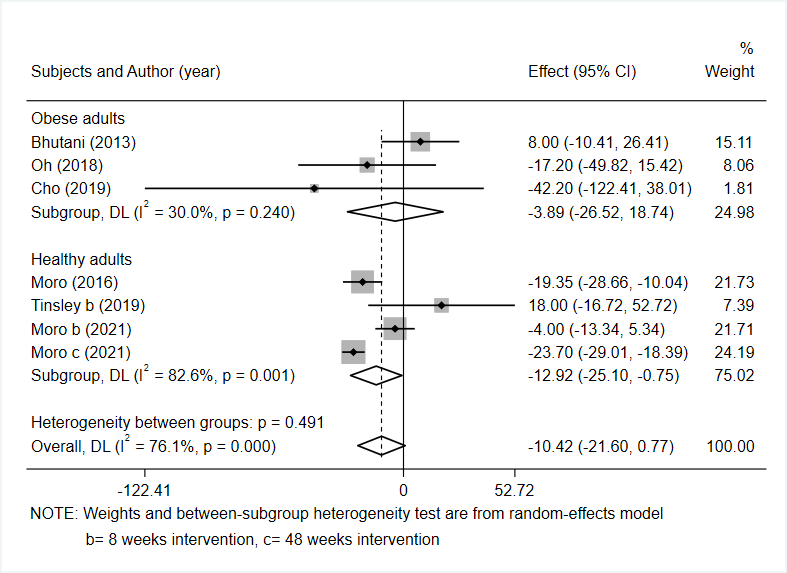


**Supplementary Figure 14.** Forest plot of subgroup analysis for the effects on TG in groups with different subject types.


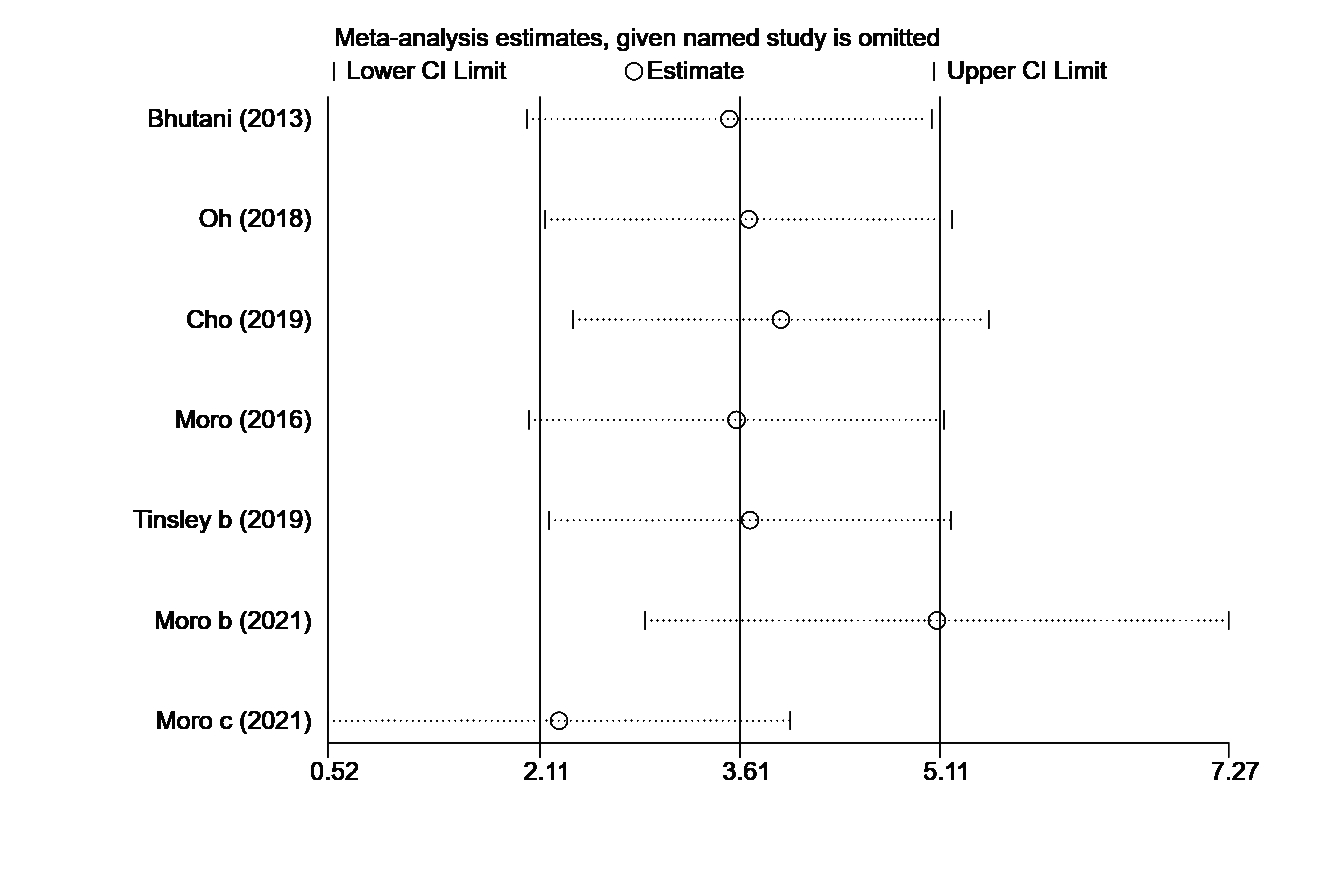


**Supplementary Figure 15.** Sensitivity analysis outcome for HDL-C.


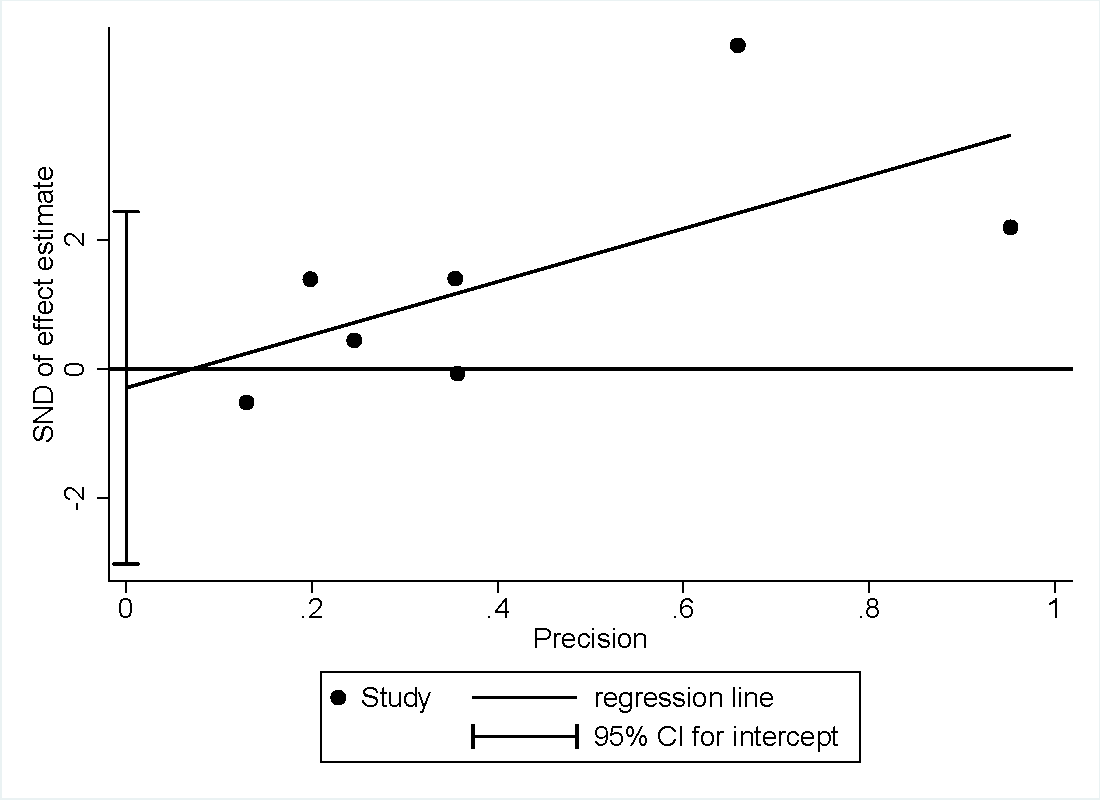


**Supplementary Figure 16.** Egger’s test plot for HDL-C.


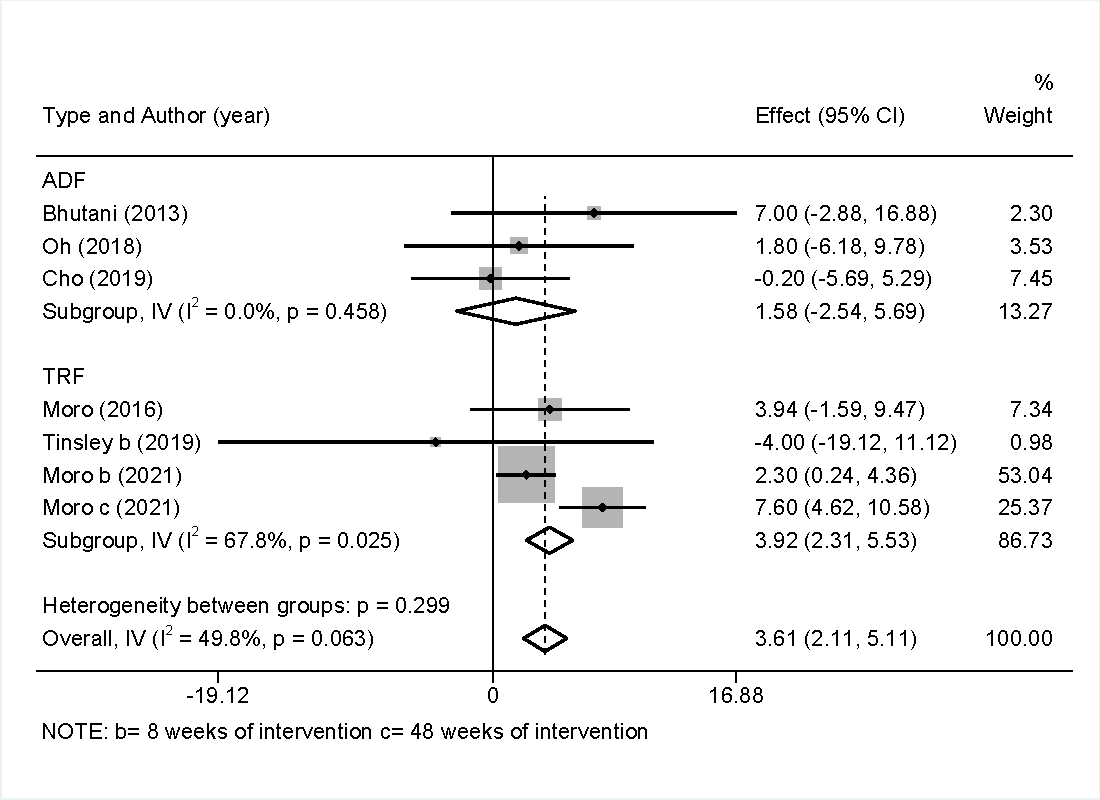


**Supplementary Figure 17.** Forest plot of subgroup analysis for the effects on HDL-C in groups with different IF subtypes.


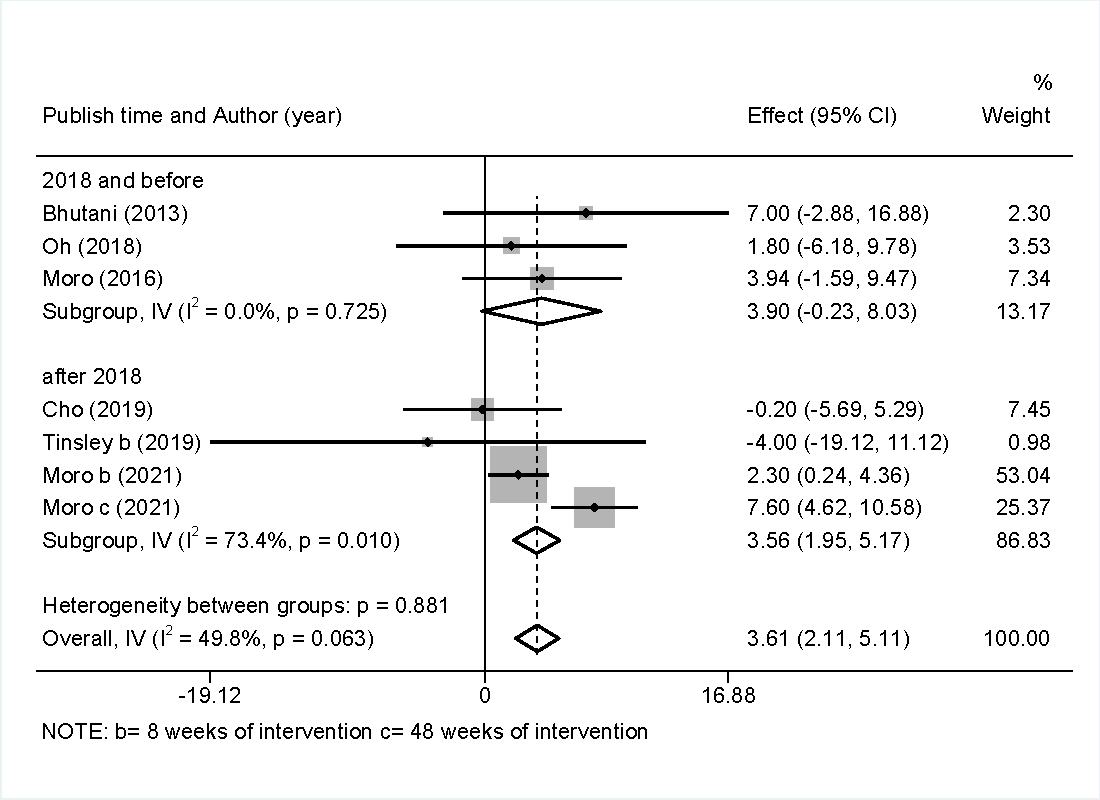


**Supplementary Figure 18.** Forest plot of subgroup analysis for the effects on HDL-C in groups with different publication time.


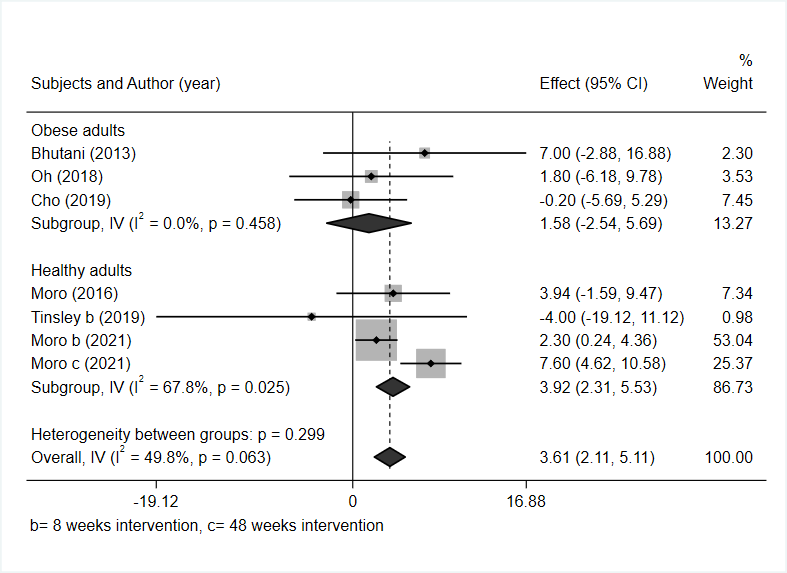


**Supplementary Figure 19.** Forest plot of subgroup analysis for the effects on HDL-C in groups with different subject types.

## Supplementary Tables

**Supplementary Table 1.** PRISMA checklist.

| **Section and Topic** | **Item #** | **Checklist item** | **Location where item is reported** |
| --- | --- | --- | --- |
| **TITLE** | | |  |
| Title | 1 | Identify the report as a systematic review. | Pg1 |
| **ABSTRACT** | | |  |
| Abstract | 2 | See the PRISMA 2020 for Abstracts checklist. | Pg1 |
| **INTRODUCTION** | | |  |
| Rationale | 3 | Describe the rationale for the review in the context of existing knowledge. | Pg1-2 |
| Objectives | 4 | Provide an explicit statement of the objective(s) or question(s) the review addresses. | Pg2 |
| **METHODS** | | |  |
| Eligibility criteria | 5 | Specify the inclusion and exclusion criteria for the review and how studies were grouped for the syntheses. | Pg2-3 |
| Information sources | 6 | Specify all databases, registers, websites, organisations, reference lists and other sources searched or consulted to identify studies. Specify the date when each source was last searched or consulted. | Pg2 |
| Search strategy | 7 | Present the full search strategies for all databases, registers, and websites, including any filters and limits used. | Pg3, Tab S2 |
| Selection process | 8 | Specify the methods used to decide whether a study met the inclusion criteria of the review, including how many reviewers screened each record and each report retrieved, whether they worked independently, and if applicable, details of automation tools used in the process. | Pg2-3 |
| Data collection process | 9 | Specify the methods used to collect data from reports, including how many reviewers collected data from each report, whether they worked independently, any processes for obtaining or confirming data from study investigators, and if applicable, details of automation tools used in the process. | Pg3 |
| Data items | 10a | List and define all outcomes for which data were sought. Specify whether all results that were compatible with each outcome domain in each study were sought (e.g. for all measures, time points, analyses), and if not, the methods used to decide which results to collect. | Pg3 |
|  | 10b | List and define all other variables for which data were sought (e.g. participant and intervention characteristics, funding sources). Describe any assumptions made about any missing or unclear information. | Pg3 |
| Study risk of bias assessment | 11 | Specify the methods used to assess risk of bias in the included studies, including details of the tool(s) used, how many reviewers assessed each study and whether they worked independently, and if applicable, details of automation tools used in the process. | Pg3 |
| Effect measures | 12 | Specify for each outcome the effect measure(s) (e.g. risk ratio, mean difference) used in the synthesis or presentation of results. | Pg3 |
| Synthesis methods | 13a | Describe the processes used to decide which studies were eligible for each synthesis (e.g. tabulating the study intervention characteristics and comparing against the planned groups for each synthesis (item #5)). | Pg4 |
|  | 13b | Describe any methods required to prepare the data for presentation or synthesis, such as handling of missing summary statistics, or data conversions. | Pg4 |
|  | 13c | Describe any methods used to tabulate or visually display results of individual studies and syntheses. | Pg4 |
|  | 13d | Describe any methods used to synthesize results and provide a rationale for the choice(s). If meta-analysis was performed, describe the model(s), method(s) to identify the presence and extent of statistical heterogeneity, and software package(s) used. | Pg4 |
|  | 13e | Describe any methods used to explore possible causes of heterogeneity among study results (e.g. subgroup analysis, meta-regression). | Pg4 |
|  | 13f | Describe any sensitivity analyses conducted to assess robustness of the synthesized results. | Pg4 |
| Reporting bias assessment | 14 | Describe any methods used to assess risk of bias due to missing results in a synthesis (arising from reporting biases). | Pg4 |
| Certainty assessment | 15 | Describe any methods used to assess certainty (or confidence) in the body of evidence for an outcome. | Pg4 |
| **RESULTS** | | |  |
| Study selection | 16a | Describe the results of the search and selection process, from the number of records identified in the search to the number of studies included in the review, ideally using a flow diagram. | Pg3-4, Fig1 |
|  | 16b | Cite studies that might appear to meet the inclusion criteria, but which were excluded, and explain why they were excluded. | Pg4, Fig1 |
| Study characteristics | 17 | Cite each included study and present its characteristics. | Pg4-12, Tab1 |
| Risk of bias in studies | 18 | Present assessments of risk of bias for each included study. | Pg12, Fig2 |
| Results of individual studies | 19 | For all outcomes, present, for each study: (a) summary statistics for each group (where appropriate) and (b) an effect estimate and its precision (e.g. confidence/credible interval), ideally using structured tables or plots. | Pg13-20 |
| Results of syntheses | 20a | For each synthesis, briefly summarise the characteristics and risk of bias among contributing studies. | Pg13-20 |
|  | 20b | Present results of all statistical syntheses conducted. If meta-analysis was done, present for each the summary estimate and its precision (e.g. confidence/credible interval) and measures of statistical heterogeneity. If comparing groups, describe the direction of the effect. | Pg13-20, Fig 3, 5-9, Tab S3 |
|  | 20c | Present results of all investigations of possible causes of heterogeneity among study results. | Pg13-20 |
|  | 20d | Present results of all sensitivity analyses conducted to assess the robustness of the synthesized results. | Pg13-20, Fig 4 |
| Reporting biases | 21 | Present assessments of risk of bias due to missing results (arising from reporting biases) for each synthesis assessed. | Pg13-20, Fig S2, S4, S7, S11 |
| Certainty of evidence | 22 | Present assessments of certainty (or confidence) in the body of evidence for each outcome assessed. | Pg13-20 |
| **DISCUSSION** | | |  |
| Discussion | 23a | Provide a general interpretation of the results in the context of other evidence. | Pg20-21 |
|  | 23b | Discuss any limitations of the evidence included in the review. | Pg21 |
|  | 23c | Discuss any limitations of the review processes used. | Pg21 |
|  | 23d | Discuss implications of the results for practice, policy, and future research. | Pg21, Fig S1, S3, S6, S10 |
| **OTHER INFORMATION** | | |  |
| Registration and protocol | 24a | Provide registration information for the review, including register name and registration number, or state that the review was not registered. | Pg2 |
|  | 24b | Indicate where the review protocol can be accessed, or state that a protocol was not prepared. | Pg2 |
|  | 24c | Describe and explain any amendments to information provided at registration or in the protocol. | Pg2 |
| Support | 25 | Describe sources of financial or non-financial support for the review, and the role of the funders or sponsors in the review. | Pg22 |
| Competing interests | 26 | Declare any competing interests of review authors. | Pg22 |
| Availability of data, code and other materials | 27 | Report which of the following are publicly available and where they can be found: template data collection forms; data extracted from included studies; data used for all analyses; analytic code; any other materials used in the review. | Pg22 |

**Supplementary Table 2.** Search strategies (example for 3 databases).

| **PubMed** | **Search terms for query** | **Results** |
| --- | --- | --- |
| #1 | (((((((((((((("Exercise"[Mesh]) OR (Exercises[MeSH Terms])) OR (Physical Activity[MeSH Terms])) OR (Physical Activities[MeSH Terms])) OR (Physical Exercise[MeSH Terms])) OR (Physical Exercises[MeSH Terms])) OR (Acute Exercise[MeSH Terms])) OR (Acute Exercises[MeSH Terms])) OR (Isometric Exercises[MeSH Terms])) OR (Isometric Exercise[MeSH Terms])) OR (Aerobic Exercise[MeSH Terms])) OR (Aerobic Exercises[MeSH Terms])) OR (Exercise Training[MeSH Terms])) OR (Exercise Trainings[MeSH Terms]) OR ((((((((((((("Resistance Training"[Mesh]) OR (Strength Training[MeSH Terms])) OR (Weight-Lifting Strengthening Program[MeSH Terms])) OR (Weight Lifting Strengthening Program[MeSH Terms])) OR (Weight-Lifting Strengthening Programs[MeSH Terms])) OR (Weight-Lifting Exercise Program[MeSH Terms])) OR (Weight-Lifting Exercise Programs[MeSH Terms])) OR (Weight Lifting Exercise Program[MeSH Terms])) OR (Weight-Bearing Strengthening Program[MeSH Terms])) OR (Weight Bearing Strengthening Program[MeSH Terms])) OR (Weight-Bearing Strengthening Programs[MeSH Terms])) OR (Weight-Bearing Exercise Program[MeSH Terms])) OR (Weight Bearing Exercise Program[MeSH Terms])) OR (Weight-Bearing Exercise Programs[MeSH Terms])) OR ((((((((((((("High-Intensity Interval Training"[Mesh]) OR (High-Intensity Interval Trainings[MeSH Terms])) OR (High Intensity Interval Training[MeSH Terms])) OR (Interval Training, High-Intensity[MeSH Terms])) OR (Interval Trainings, High-Intensity[MeSH Terms])) OR (Training, High-Intensity Interval[MeSH Terms])) OR (Trainings, High-Intensity Interval[MeSH Terms])) OR (High-Intensity Intermittent Exercise[MeSH Terms])) OR (Exercise, High-Intensity Intermittent[MeSH Terms])) OR (Exercises, High-Intensity Intermittent[MeSH Terms])) OR (High-Intensity Intermittent Exercises[MeSH Terms])) OR (Sprint Interval Training[MeSH Terms])) OR (Sprint Interval Trainings[MeSH Terms])) Filters: from 1945/1/1 - 2021/12/31 | 258897 |
| #2 | ((((((((("Fasting"[Mesh]) OR (Intermittent Fasting[MeSH Terms])) OR (Intermittent Fastings[MeSH Terms])) OR (Hunger Strike[MeSH Terms])) OR (Hunger Strikes[MeSH Terms])) OR (Time Restricted Feeding[MeSH Terms])) OR (Time Restricted Feedings[MeSH Terms])) OR ((((5:2 fasting[Text Word]) OR (5:2 fasting[Title/Abstract])) OR (5:2 Intermittent fasting[Title/Abstract])) OR (5:2 Intermittent fasting[Text Word]))) OR ((((TRF[Title/Abstract]) OR (time restricted fasting[Title/Abstract])) OR (TRF[Text Word])) OR (time restricted fasting[Text Word]))) OR ((((alternate-day fasting[Title/Abstract]) OR (ADF[Title/Abstract])) OR (alternate-day fasting[Text Word])) OR (ADF[Text Word])) Filters: from 1945/1/1 - 2021/12/31 | 42804 |
| #3 | (#1) AND (#2) | 997 |
| #4 | ((((((((((((("Metabolic Syndrome"[Mesh]) OR (Metabolic Syndromes[MeSH Terms])) OR (Metabolic Syndrome X[MeSH Terms])) OR (Insulin Resistance Syndrome X[MeSH Terms])) OR (Metabolic X Syndrome[MeSH Terms])) OR (Syndrome, Metabolic X[MeSH Terms])) OR (Dysmetabolic Syndrome X[MeSH Terms])) OR (Reaven Syndrome X[MeSH Terms])) OR (Metabolic Cardiovascular Syndrome[MeSH Terms])) OR (Cardiovascular Syndrome, Metabolic[MeSH Terms])) OR (Cardiovascular Syndromes, Metabolic[MeSH Terms])) OR (Cardiometabolic Syndrome[MeSH Terms])) OR (Cardiometabolic Syndromes[MeSH Terms])) OR ((("Metabolic Diseases"[Mesh]) OR (Metabolic Disease[MeSH Terms])) OR (Thesaurismosis[MeSH Terms])) Filters: from 1945/1/1 - 2021/12/31 | 1096445 |
| #5 | (#3) AND (#4) | 347 |

| **Web of Science** | **Search terms for query** | **Results** |
| --- | --- | --- |
| #1 | TI=(Exercise OR Exercises OR Physical Activity OR Activities, Physical OR Exercise, Physical OR Exercises, Physical OR Acute Exercise OR Acute Exercises OR Exercise, Isometric OR Exercises, Isometric OR Exercise, Aerobic OR Aerobic Exercises OR Exercise Training OR Exercise Trainings OR Resistance Training OR Strength Training OR Weight-Lifting Strengthening Program OR Weight Lifting Strengthening Program OR Weight-Lifting Exercise Programs OR Weight Lifting Exercise Program OR Weight-Bearing Strengthening Program OR Weight Bearing Strengthening Program OR Weight-Bearing Strengthening Programs OR Weight-Bearing Exercise Program OR Weight Bearing Exercise Program OR Weight-Bearing Exercise Programs OR High-Intensity Interval Training OR High-Intensity Interval Trainings OR High Intensity Interval Training OR Interval Training, High-Intensity OR Interval Trainings, High-Intensity OR Training, High-Intensity Interval OR Trainings, High-Intensity Interval OR High-Intensity Intermittent Exercise OR Exercise, High-Intensity Intermittent OR Exercises, High-Intensity Intermittent OR High-Intensity Intermittent Exercises OR Sprint Interval Training OR Sprint Interval Trainings)  Timespan: 1950-01-01 to 2021-12-31 | 297446 |
| #2 | TI=(Fasting OR Intermittent Fasting OR Intermittent Fastings OR Hunger Strike OR Hunger Strikes OR Time Restricted Feeding OR Time Restricted Feedings OR 5:2 fasting OR 5:2 Intermittent fasting OR TRF OR time restricted fasting OR alternate-day fasting OR ADF)  Timespan: 1950-01-01 to 2021-12-31 | 180934 |
| #3 | #1 AND #2 | 816 |
| #4 | TI=(Metabolic Syndrome OR Metabolic Syndromes OR Metabolic Syndrome X OR Insulin Resistance Syndrome X OR Metabolic X Syndrome OR Syndrome, Metabolic X OR Dysmetabolic Syndrome X OR Reaven Syndrome X OR Metabolic Cardiovascular Syndrome OR Cardiovascular Syndrome, Metabolic OR Cardiovascular Syndromes, Metabolic OR Cardiometabolic Syndrome OR Cardiometabolic Syndromes OR Metabolic Diseases OR Metabolic Disease OR Thesaurismosis)  Timespan: 1950-01-01 to 2021-12-31 | 57689 |
| #5 | #3 AND #4 | 3 |

| **Cochrane library** | **Search terms for query** | **Results** |
| --- | --- | --- |
| #1 | MeSH descriptor: [Exercise] explode all trees | 27342 |
| #2 | (Exercises):ab,ti,kw OR (Physical Activity):ab,ti,kw OR (Activities, Physical):ab,ti,kw OR (Activity, Physical):ab,ti,kw OR (Physical Activities):ab,ti,kw OR (Exercise, Physical):ab,ti,kw OR (Exercises, Physical):ab,ti,kw OR (Physical Exercise):ab,ti,kw OR (Physical Exercises):ab,ti,kw OR (Acute Exercise):ab,ti,kw OR (Acute Exercises):ab,ti,kw OR (Exercise, Acute):ab,ti,kw OR (Exercises, Acute):ab,ti,kw OR (Exercise, Isometric):ab,ti,kw OR (Exercises, Isometric):ab,ti,kw OR (Isometric Exercises):ab,ti,kw OR (Isometric Exercise):ab,ti,kw OR (Exercise, Aerobic):ab,ti,kw OR (Aerobic Exercise):ab,ti,kw OR (Aerobic Exercises):ab,ti,kw OR (Exercises, Aerobic):ab,ti,kw OR (Exercise Training):ab,ti,kw OR (Exercise Trainings):ab,ti,kw OR (Training, Exercise):ab,ti,kw OR (Trainings, Exercise):ab,ti,kw | 109850 |
| #3 | (#1) OR (#2) | 117259 |
| #4 | MeSH descriptor: [Resistance Training] explode all | 3952 |
| #5 | (Training, Resistance):ab,ti,kw OR (Strength Training):ab,ti,kw OR (Training, Strength):ab,ti,kw OR (Weight-Lifting Strengthening Program):ab,ti,kw OR (Strengthening Program, Weight-Lifting):ab,ti,kw OR (Strengthening Programs, Weight-Lifting):ab,ti,kw OR (Weight Lifting Strengthening Program):ab,ti,kw OR (Weight-Lifting Strengthening Programs):ab,ti,kw OR (Weight-Lifting Exercise Program):ab,ti,kw OR (Exercise Program, Weight-Lifting):ab,ti,kw OR (Exercise Programs, Weight-Lifting):ab,ti,kw OR (Weight Lifting Exercise Program):ab,ti,kw OR (Weight-Lifting Exercise Programs):ab,ti,kw OR (Weight-Bearing Strengthening Program):ab,ti,kw OR (Strengthening Program, Weight-Bearing):ab,ti,kw OR (Strengthening Programs, Weight-Bearing):ab,ti,kw OR (Weight Bearing Strengthening Program):ab,ti,kw OR (Weight-Bearing Strengthening Programs):ab,ti,kw OR (Weight-Bearing Exercise Program):ab,ti,kw OR (Exercise Program, Weight-Bearing):ab,ti,kw OR (Exercise Programs, Weight-Bearing):ab,ti,kw OR (Weight Bearing Exercise Program):ab,ti,kw OR (Weight-Bearing Exercise Programs):ab,ti,kw | 23145 |
| #6 | #4 OR #5 | 23145 |
| #7 | MeSH descriptor: [High-Intensity Interval Training] explode all trees | 593 |
| #8 | (High Intensity Interval Training):ab,ti,kw OR (High-Intensity Interval Trainings):ab,ti,kw OR (Interval Training, High-Intensity):ab,ti,kw OR (Interval Trainings, High-Intensity):ab,ti,kw OR (Training, High-Intensity Interval):ab,ti,kw OR (Trainings, High-Intensity Interval):ab,ti,kw OR (High-Intensity Intermittent Exercise):ab,ti,kw OR (Exercise, High-Intensity Intermittent):ab,ti,kw OR (Exercises, High-Intensity Intermittent):ab,ti,kw OR (High-Intensity Intermittent Exercises):ab,ti,kw OR (Sprint Interval Training):ab,ti,kw OR (Sprint Interval Trainings):ab,ti,kw | 3278 |
| #9 | #7 OR #8 | 3278 |
| #10 | #3 OR #6 #9 | 117317 |
| #11 | MeSH descriptor: [Fasting] explode all trees | 3385 |
| #12 | (Intermittent Fasting):ab,ti,kw OR (Fasting, Intermittent):ab,ti,kw OR (Intermittent Fastings):ab,ti,kw OR (Hunger Strike):ab,ti,kw OR (Hunger Strikes):ab,ti,kw OR (Strike, Hunger):ab,ti,kw OR (Strikes, Hunger):ab,ti,kw OR (Time Restricted Feeding):ab,ti,kw OR (Feeding, Time Restricted):ab,ti,kw OR (Time Restricted Feedings):ab,ti,kw | 684 |
| #13 | #11 OR #12 | 3988 |
| #14 | #10 AND #13 | 454 |
| #15 | MeSH descriptor: [Metabolic Syndrome] explode all trees | 1990 |
| #16 | (Metabolic Syndromes):ab,ti,kw OR (Syndrome, Metabolic):ab,ti,kw OR (Syndromes, Metabolic):ab,ti,kw OR (Metabolic Syndrome X):ab,ti,kw OR (Insulin Resistance Syndrome X):ab,ti,kw OR (Syndrome X, Metabolic):ab,ti,kw OR (Syndrome X, Insulin Resistance):ab,ti,kw OR (Metabolic X Syndrome):ab,ti,kw OR (Syndrome, Metabolic X):ab,ti,kw OR (X Syndrome, Metabolic):ab,ti,kw OR (Dysmetabolic Syndrome X):ab,ti,kw OR (Syndrome X, Dysmetabolic):ab,ti,kw OR (Reaven Syndrome X):ab,ti,kw OR (Syndrome X, Reaven):ab,ti,kw OR (Metabolic Cardiovascular Syndrome):ab,ti,kw OR (Cardiovascular Syndrome, Metabolic):ab,ti,kw OR (Cardiovascular Syndromes, Metabolic):ab,ti,kw OR (Syndrome, Metabolic Cardiovascular):ab,ti,kw OR (Cardiometabolic Syndrome):ab,ti,kw OR (Cardiometabolic Syndromes):ab,ti,kw OR (Syndrome, Cardiometabolic):ab,ti,kw OR (Syndromes, Cardiometabolic):ab,ti,kw | 10258 |
| #17 | #15 OR #16 | 10258 |
| #18 | #14 AND #17 | 29 |

**Supplementary Table 3.** Characteristics of the included trials.

| **Trial** | **Region** | **Subjects** | **Sample Size** | | **MeanBM (kg)** | | **MeanBMI (kg/m^2^)** | | **MeanWC (cm)** | | **MeanHDL-C (mg/dl)** | | **MeanTG (mg/dl)** | | **MeanFBG (mg/dl)** | | **MeanSBP (mmHg)** | | **MeanDBP (mmHg)** | | **Physical Activity Level** | **Mean Age** | **IF**  **Intervention** | **MVPA**  **Intervention** | **Study Duration** | **Outcomes** | **Results** |
| --- | --- | --- | --- | --- | --- | --- | --- | --- | --- | --- | --- | --- | --- | --- | --- | --- | --- | --- | --- | --- | --- | --- | --- | --- | --- | --- | --- |
|  |  |  | **IG** | **CG** | **IG** | **CG** | **IG** | **CG** | **IG** | **CG** | **IG** | **CG** | **IG** | **CG** | **IG** | **CG** | **IG** | **CG** | **IG** | **CG** |  |  |  |  |  |  |  |
| Bhutani et al. (2013) | America | obese males and females | 18 | 24.0(PA)  25.0(IF) | 91.0 | 93.0(PA)  94.0(IF) | 35 | 35.0(PA)  35.0(IF) | 96.0 | 98.0(PA)  100.0(IF) | 50.0 | 51.0(PA)  49.0(IF) | 77.0 | 74.0(PA)  81.0(IF) | 94.0 | 92.0(PA)  98.0(IF) | 113.0 | 113.0(PA)  124.0(IF) | 76 | 76(PA)  82(IF) | Lightly active (i.e., <3 h/week of light intensity exercise at 2.5-4.0 metabolic equivalents (METs) for 3 months prior to the study) | 43.3 | ADF: (week 1-4) subjects consumed 25% of their baseline energy needs on the “fast day” (24 h). Fast day meals were consumed between 12.00 pm and 2.00 pm. (week 8-12) subjects continued with the ADF regimen, each subject met with a dietician at the beginning of each week to learn how to maintain the ADF regimen on his or her own at home. | ET: subjects participated in a moderate intensity exercise program three times per week for 12 weeks. Each training session began with a 5-min warm-up period, and ended with a 5-min cool-down. | 12 weeks | BM  FBG  TG  HDL-C  SBP  DBP | Compared to non-intervention controls:  BM ↓  FBG ↓  TG -  HDL-C ↑  SBP -  DBP - |
| Oh et al. (2018) | Korea | overweight and obese adults | 12 | 10(PA)  13(IF) | 74.0 | 83.2(PA)  74.1(IF) | 27.5 | 28.3(PA)  27.6(IF) | 90.5 | 95.5(PA)  91.6(IF) | 48.0 | 44.8(PA)  58.5(IF) | 133.4 | 142.7(PA)  108.8(IF) | 98.0 | 93.0(PA)  90.0(IF) | 120.8 | 125.3(PA)  119.6(IF) | 80.3 | 83.5(PA)  78.1(IF) | NR | 36.6 | ADF：25% of normal energy intake (400–500 kcal) between 12 pm and 2 pm on 3 nonconsecutive days per week, with ad libitum eating on the remaining 4 days of the week. | RT and AE: at least 3 times/week for 8 weeks, one session of exercise included the following components: 1) 5 min of warm-up; 2) 40 min of resistance training; 3) 20 min of aerobic exercise; 4) 5 min of cool-down. | 8 weeks | BM  FBG  TG  HDL-C  SBP  DBP | Compared to non-intervention controls:  BM ↓  FBG ↓  TG ↓  HDL-C ↑  SBP -  DBP - |
| Cho et al. (2019) | Korea | overweight or obese adults | 28 | 24(PA)  26(IF) | 78.2 | 74.2(PA)  74.6(IF) | 28.0 | 26.9(PA)  27.8(IF) | NR | NR | 46.5 | 41.5(PA)  47.9(IF) | 122.0 | 208.5(PA)  117.3(IF) | 94.5 | 90.3(PA)  96.2(IF) | NR | NR | NR | NR | 1097.7 MET-min/wk (IG)  862.2 MET-min/wk (PA)  1230.0 MET-min/wk (IF) | 36.5 | ADF: participants consumed 25% of their daily recommended energy intake (one meal between 12 PM and 2 PM, approximately 500 kcal) on each “fast day” (24 h), and consumed food ad libitum on each “feed day” (24 h). The “fast day” and “feed day” were repeated every other day, and the “fast day” occurred 3 days per week. | RT and AE: at least 3 times/week for 8 weeks, each exercise session began with 5 min of warm-up and ended with 5 min of cool-down. Resistance training was per-formed for 40 min. Aerobic exercise was performed for 20 min. | 8 weeks | BM  FBG  TG  HDL-C | Compared to non-intervention controls:  BM ↓  FBG -  TG ↓  HDL-C - |
| Moro et al. (2016) | Italy | healthy resistance-trained males | 17 | 17 | 84.0 | 85.3 | NR | NR | NR | NR | 54.1 | 53.3 | 123.8 | 137.7 | 96.6 | 95.2 | NR | NR | NR | NR | Subjects must have performed resistance training continuously for at least 5 years (training 3–5 days/week) | 29.2 | TRF: subjects consumed 100 % of their energy needs divided into three meals consumed at 1 p.m., 4 p.m. and 8 p.m., and fasted for the remaining 16 h per 24-h period. ND group ingested their caloric intake as three meals consumed at 8 a.m., 1 p.m. and 8 p.m. | RT: Training consisted of 3 weekly sessions performed on non-consecutive days for 8 weeks. | 8 weeks | BM  FBG  TG  HDL-C | Compared to the MVPA intervention group:  BM NR  FBG ↓  TG ↓  HDL-C ↑ |
| Tinsley et al. (2019) | America | healthy females have prior RT experience | 13 | 14 | 63.8 | 64.7 | NR | NR | NR | NR | 64.0 | 69.0 | 88.0 | 83.0 | 89.0 | 93.0 | 97.0 | 94.0 | 68.0 | 66.0 | Subjects were required to have prior RT experience, defined as reporting ≥1 y of RT at a frequency of 2 to 4 sessions per week. | 22.9 | TRF: subjects consumed all calories between 1200 h and 2000 h each day, and others consumed breakfast after waking and to continue to eat at self-selected intervals throughout the remainder of the day. | RT: 8 weeks of supervised RT, RT sessions were completed on 3 nonconsecutive days each week (i.e., Mondays, Wednesdays, and Fridays), and 2 different upper- and lower-body sessions were alternated. | 4/8 weeks | BM  FBG  TG  HDL-C  SBP  DBP | Compared to the MVPA intervention group:  BM -  FBG -  TG -  HDL-C -  SBP -  DBP - |
| Moro et al. (2021) | Italy | healthy subjects | 10 | 10 | 83.2 | 84.6 | NR | NR | NR | NR | 53.7 | 53.6 | 123.2 | 122.1 | 95.1 | 95.5 | NR | NR | NR | NR | Subjects were required to have a regular practice of resistance training for at least 5 continuously years. | NR | TRF: subjects consumed the total caloric needs divided into three meals eaten in an 8-h window (~1 p.m., 4 p.m. and 8 p.m.), and fasted for the remaining 16 h Calories distribution was 40% at breakfast, 25% at lunch, and 35% at dinner. | RT: subjects maintained a thrice-weekly training schedule. Training intensity fluctuated during the experimental period between 75% and 90% of 1-RM to alternate strength and hypertrophy training cycles. Training sessions were performed between 4:00 and 6:00 p.m. | 8/48 weeks | BM  FBG  TG  HDL-C | Compared to the MVPA intervention group:  BM ↓  FBG ↓  TG ↓  HDL-C ↑ |
| Martínez-Rodríguez et al. (2021) | Spain | active women | 7 | 7 | 59.9 | 58.9 | NR | NR | 68.2 | 68.5 | NR | NR | NR | NR | NR | NR | NR | NR | NR | NR | Active women (perform physical exercises or general vigorous training at least 3 times a week on non-consecutive days.) | 27 | TRF: Participants were instructed to not eat in <14 h of the day before, and consume breakfast as soon as possible after waking and to continue to eat following the diet intervals throughout the remainder of the day. | HIIT: 3 times per week, with the training sessions separated by 48 h. HIIT sessions lasted 40 min. The training session consisted of 3×10 repetitions of 30 s of aerobic exercises all out interspersed by 30 s of rest. | 16 weeks | BM | Compared to the MVPA intervention group:  BM ↓ |
| Tinsley et al. (2017) | America | Generally healthy and recreationally active men | 14 | 14 | 87.4 | 79.0 | NR | NR | NR | NR | NR | NR | NR | NR | NR | NR | NR | NR | NR | NR | Young recreationally active males | 22.5 | TRF: subjects consumed all calories in any four-hour window between 4 p.m. and midnight. The number of calories and specific foods were not limited. | RT: three nonconsecutive days per week. Subjects alternated between upper and lower body workouts. Four sets of each exercise were performed and a 90-second rest period between sets was assigned. | 4/8 weeks | BM | Compared to the MVPA intervention group:  BM ↓ |
| Stratton et al. (2020) | America | recreationally active men | 16 | 16 | 82.0 | 83.3 | NR | NR | NR | NR | NR | NR | NR | NR | NR | NR | NR | NR | NR | NR | All participants were categorized as recreationally active, which was operationally defined as engaging in resistance training 2–4 times per week for the past six months. | 22.7 | TRF: with all calorie and macronutrient consumption occurring within an 8 h period each day and a prescribed 25% caloric deficit. | RT: full body sessions performed three times per week. The leg press and bench press were performed in all sessions, followed by a horizontal rowing exercise, a shoulder exercise, quadricep and hamstring dominant exercises performed in supersetted fashion, and tricep and bicep exercises performed as a superset. | 4 weeks | BM | Compared to the MVPA intervention group:  BM ↓ |
| Tovar et al. (2021) (52) | America | healthy, endurance trained male runners | 10 | 9 | 73.0 | 73.0 | NR | NR | NR | NR | NR | NR | NR | NR | NR | NR | NR | NR | NR | NR | NR | 28.7 | TRF: a 16/8 time-restricted feeding protocol. Subjects were required to consume all meals in the same 8-h period of their choosing daily. | Following the substrate utilization test and 10 min of rest, subjects completed a 10 km running time trial on the treadmill as quickly as possible and were instructed to treat the exercise as competitive race. Subjects then walked at 4.8 km·h−1 for 5 min, followed by 10 min of seated rest. | 4 weeks | BM | Compared to the MVPA intervention group:  BM - |
| Correia et al. (2021) (53) | Portugal | well-trained young men | 9 | 9 | 73.6 | 73.5 | NR | NR | NR | NR | NR | NR | NR | NR | NR | NR | NR | NR | NR | NR | participated in power-sports training (training frequency of at least 3 times per week). | 22.4 | TRF: a 16/8 time-restricted feeding protocol. Two or three meals of meals of ad libitum food intake during an 8-h period (between 1 and 9 p.m.). The remaining 16 hours per 24-h time period constituted the fasting period during which participants only were allowed to consume drinks without caloric additives. | Subjects were asked to continue their habitual training throughout the study. | 4 weeks | BM | Compared to the MVPA intervention group:  BM - |

Abbreviations: BM: Body mass; BMI: Body mass index; WC: Waist circumference; TC: Total cholesterol; HDL-C: High-density lipoprotein cholesterol; LDL-C: Low-density lipoprotein cholesterol; TG: Triglycerides; FBG: Fasting blood glucose; IG: Intervention group; CG: Control group; SBP: Systolic blood pressure; DBP: Diastolic blood pressure; IF: Intermittent fasting; MVPA: Moderate-to-vigorous physical activities; ADF: Alternate day fasting; ET: Endurance training; RT: Resistance training; AE: Aerobic Exercise; HIIT: High-intensity interval training; NR = not reported.

**Supplementary Table 4.** Results of Meta-analysis (Combined intervention versus intermittent fasting alone)

| Outcomes | References | WMD | 95% CI | *P*-value | Effect Model | Heterogeneity | |
| --- | --- | --- | --- | --- | --- | --- | --- |
|  |  |  |  |  |  | *I^2^* | *P*-value |
| BM | Bhutani et al. (2013)  Oh et al. (2018)  Cho et al. (2019) | -0.43 | -6.69 to 5.82 | 0.893 | Fixed-effects model | 0.0 | 0.516 |
| FBG | Bhutani et al. (2013)  Oh et al. (2018)  Cho et al. (2019) | -3.72 | -8.36 to 0.92 | 0.116 | Fixed-effects model | 0.0 | 0.513 |
| TG | Bhutani et al. (2013)  Oh et al. (2018)  Cho et al. (2019) | -9.59 | -28.63 to 9.46 | 0.324 | Fixed-effects model | 44.4 | 0.166 |
| HDL-C | Bhutani et al. (2013)  Oh et al. (2018)  Cho et al. (2019) | 1.459 | -6.46 to 9.56 | 0.724 | Random-effects model | 66.9 | 0.049 |
| SBP | Bhutani et al. (2013)  Oh et al. (2018) | -5.83 | -12.51 to 0.85 | 0.087 | Fixed-effects model | 32.5 | 0.223 |
| DBP | Bhutani et al. (2013)  Oh et al. (2018) | -2.54 | -7.05 to 1.97 | 0.270 | Fixed-effects model | 0.0 | 0.331 |

Abbreviations: BM: Body mass; FBG: Fasting blood glucose; TG: Triglycerides; HDL-C: High-density lipoprotein cholesterol; SBP: Systolic blood Pressure; DBP: Diastolic blood pressure; WMD: Weighted Mean Difference; CI: Confidence Interval.
